# *SLC29A1*/ENT1 and *SLC29A3*/ENT3 differentially regulate autophagy

**DOI:** 10.1080/15548627.2026.2639407

**Published:** 2026-03-05

**Authors:** Bhawana Bissa, Tejinder Kaur, Arnav Joshi, Rajgopal Govindarajan

**Affiliations:** a Division of Pharmaceutics and Pharmacology, College of Pharmacy, The Ohio State University, Columbus, OH, USA; b Translational Therapeutics, Ohio State University Comprehensive Cancer Center, The Ohio State University, Columbus, OH, USA

**Keywords:** Adenosine, AMPK, autophagy, nucleoside, transporter, ENT

## Abstract

Despite the well-established role of equilibrative nucleoside transporters (ENTs) in salvaging nucleosides for DNA synthesis, the presence of multiple ENT subfamilies within a single genome suggests putative, non-redundant functions in maintaining cellular homeostasis. In this study, we demonstrate that, in contrast to endolysosomal *SLC29A3*/ENT3, which promotes macroautophagy/autophagy, cell surface-localized *SLC29A1*/ENT1 is capable of inhibiting autophagy by suppressing PRKAA/AMPK phosphorylation. Consistent with this, silencing *SLC29A1* induces autophagy, whereas silencing *SLC29A3* suppresses it. Treatment with adenosine (Ado), a shared substrate of *SLC29A1* and *SLC29A3*, triggers PRKAA/AMPK phosphorylation and autophagy in a concentration-dependent manner. This effect is PRKAA-dependent, as Ado fails to induce autophagy in *prkaa*-null cells. Mechanistically, elevated *SLC29A1* expression promotes increased efflux and decreased intracellular retention of Ado, thereby attenuating PRKAA/AMPK activation and autophagic flux. However, this effect is contingent upon the metabolic state of the cells. Importantly, *SLC29A1*’s regulatory effect is tied to its transport function, as pharmacological inhibition of *SLC29A1* transport enhances intracellular Ado accumulation, PRKAA/AMPK phosphorylation, and autophagy. Unlike *SLC29A3*, which modulates the MTOR pathway, *SLC29A1* does not affect MTOR signaling. Instead, it promotes BECN1-BCL2 interaction, thereby inhibiting autophagosome formation. Notably, autophagy itself differentially regulates *SLC29A1* and *SLC29A3* expression, with compensatory upregulation observed when either is modulated. Finally, *slc29a1*^−/−^ and *slc29a3*^−/−^ mice display autophagic proficiency and deficiency, respectively. These findings underscore a dynamic and reciprocal regulatory relationship between *SLC29A1* and *SLC29A3* in autophagy, offering new avenues for therapeutic modulation in autophagy-related disorders.

## Introduction

Autophagy is a conserved intracellular degradation pathway that delivers cytoplasmic constituents to the lysosome. Mechanistically, three distinct forms of autophagy have been identified: macroautophagy/autophagy, chaperone-mediated autophagy, and microautophagy [1]. The precise regulation of autophagy is essential for cellular adaptation to nutrient availability and for maintaining metabolic homeostasis and viability [2–4]. Recent advances have highlighted autophagy’s involvement in diverse physiological and pathological processes, including development, aging, starvation adaptation, and tumor suppression. Nutrient depletion, such as glucose or amino acid starvation, activates autophagy through AMP-activated protein kinase (AMPK) and MTOR (mechanistic target of rapamycin kinase) signaling pathways, which regulate catabolic and anabolic processes, respectively [5–10].

While much of the research has focused on the role of carbohydrates, proteins, and lipids in autophagy regulation, the contribution of nucleotides and their degradation products remains less understood [11]. Interestingly, autophagy itself plays a critical role in maintaining intracellular nucleotide pools and providing precursors for DNA synthesis and repair [12, 13]. RNA degradation during nitrogen starvation in yeast [14–16], as well as RNA catabolism in plants and animals, has been shown to occur via autophagy [17–20]. Not only do RNA and RNA associated proteins act as autophagic cargo but also as regulators of the process [18–20]. Moreover, extracellular nucleotides such as ATP can induce autophagy in the central nervous system under hypoxic, ischemic, and inflammatory conditions [21,22]. Additionally, autophagy induction is primarily responsible for myco-bactericidal effect of ATP within human monocytes or macrophages. Furthermore, the purine nucleoside adenosine (Ado) has also been shown to promote autophagy in endothelial progenitor cells, contributing to tissue repair in diabetic ischemic ulcers [23]. Ado activates PRKAA/AMPK and promotes ACACA/ACC (acetyl-CoA carboxylase) phosphorylation [24], but its effects are blocked by inhibitors of high-affinity Ado transporters or ADK (adenosine kinase), suggesting that intracellular uptake and metabolism are essential for its regulation [25].

Despite these insights, the precise role of membrane-bound Ado transporters in autophagy regulation remains elusive. Ado, being hydrophilic (XlogP3 = −1.1), preponderantly requires nucleoside transporters to cross the cell membrane. Its cellular compartmentalization, which is intimately tied to Ado-mediated cellular processes, is tightly regulated by two major transporter families: *SLC28A*/Na^+^ -dependent concentrative nucleoside transporters/CNTs (high affinity, low capacity) and *SLC29A*/Na^+^ -independent equilibrative nucleoside transporters/ENTs (high capacity, low affinity) [26–28]. The *SLC29A*/ENTs, which include *SLC29A1*/ENT1, *SLC29A2*/ENT2, *SLC29A3*/ENT3, and *SLC29A4*/ENT4, are more widely expressed than CNTs and differ in localization and inhibitor sensitivity. *SLC29A1*, *SLC29A2*, and *SLC29A4* are localized to the plasma membrane, while *SLC29A3* is found in intracellular organelles such as mitochondria and endolysosomes [29–33]. Unlike the *SLC28A*s, *SLC29A*s are inhibited by nM (sensitive, es; SLC29A1) or μM (insensitive, ei; *SLC29A2*, *SLC29A3*) concentration of NBMPR and clinically used vasodilatory agents (i.e., dilazep and dipyridamole). *SLC29A1* inhibition by dilazep and dipyridamole has been shown to increase intracellular Ado and confer cardioprotection [34–36]. Our previous work demonstrated that *SLC29A3* regulates PRKAA/AMPK phosphorylation and autophagy through acidic pH-dependent lysosomal Ado transport [32] and *slc29a3*-deficient mice exhibit impaired autophagy and PRKAA/AMPK signaling in adult stem cells and terminally differentiated tissues [37].

In this study, we investigated the roles of cell surface-localized *SLC29A1* and endolysosomal *SLC29A3* in regulating autophagy through cellular Ado compartmentalization. We examined their effects on the PRKAA/AMPK-MTOR-ULK1 axis and BECN1-BCL2 interactions, which are critical for autophagy initiation [2, 38]. Our findings demonstrate that subcellular compartmentalization of Ado distinctly regulates autophagy through ENTs. Specifically, *SLC29A1* can inhibit autophagy by reducing PRKAA/AMPK phosphorylation and enhancing the BECN1-BCL2 interaction, whereas *SLC29A3* promotes autophagy by activating PRKAA/AMPK and suppressing MTOR signaling. We also observed reciprocal regulation of *SLC29A1* and *SLC29A3* expression, suggesting a compensatory mechanism in maintaining autophagic balance. Together, our results highlight the differential impact of *SLC29A1* and *SLC29A3* on Ado-mediated autophagy and underscore the importance of nucleoside transporter dynamics in cellular homeostasis and disease.

## Results

### Opposing roles of cell surface SLC29A1 and endolysosomal SLC29A3 in basal autophagy

We previously reported that endolysosomal *SLC29A3* promotes autophagy through the PRKAA/AMPK signaling axis [37]. To investigate the role of cell surface ENTs in autophagy, we focused on *SLC29A1*, a prototypical plasma membrane-localized nucleoside transporter, and its influence on basal autophagy. Human embryonic kidney (HEK293) cells were selected for this study due to their endogenous expression of both *SLC29A1* and *SLC29A3* at the plasma membrane and intracellular vesicles, respectively [39] (Fig. S1A, S1B), and their established use in autophagy research [40, 41].

**Figure 1.**
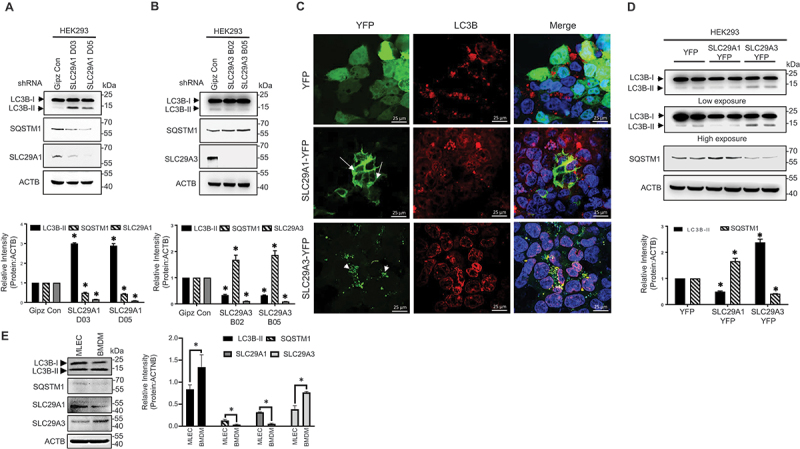
Differential regulation of autophagy by *SLC29A1* and *SLC29A3*. (A, B) HEK293 cells stably expressing control shRNA (GIPZ), *SLC29A1* shRNAs (D03, D05), or *SLC29A3* shRNAs (B02, B05) were analyzed by immunoblotting for LC3B, SQSTM1, *SLC29A1*, *SLC29A3*, and ACTB (loading control). Densitometric quantification of LC3B-II and SQSTM1 levels normalized to ACTB is shown (*n* = 3). Data are presented as mean ± SEM. *p < 0.05. (C) Immunofluorescence analysis of endogenous LC3B (red) in HEK293 cells transfected with YFP, *SLC29A1*-YFP, or *SLC29A3*-YFP. *SLC29A1*-YFP shows plasma membrane localization (green, arrows), while *SLC29A3*-YFP localizes intracellularly (green, arrowheads). Nuclei were stained with DAPI (blue). Scale bar: 25 μm. (D) HEK293 cells transiently transfected with YFP, *SLC29A1*-YFP, or *SLC29A3*-YFP were immunoblotted for LC3B and SQSTM1. ACTB served as a loading control. Densitometric analysis of LC3B-II and SQSTM1 levels normalized to ACTB is shown (*n* = 3). Data are presented as mean ± SEM. *p < 0.05. (E) Western blotting analysis of murine lung endothelial cells (MLECs) and bone marrow derived macrophages (BMDMs) for *SLC29A1*, or *SLC29A3*, LC3B, SQSTM1, and ACTB (loading control).

To assess *SLC29A1*’s role, we evaluated multiple shRNA constructs targeting *SLC29A1*. Lentiviral transduction of two constructs (D03 and D05) targeting exonic regions reduced *SLC29A1* expression by over 50% and 60%, respectively (Fig S2). Stable subclones with reduced *SLC29A1* expression were analyzed for changes in autophagy markers: MAP1LC3B/LC3B-II (LC3-I conjugated to phosphatidylethanolamine) and SQSTM1/p62 (sequestosome 1; autophagy receptor and substrate). For comparison, we included two previously established *SLC29A3* KD subclones (B03 and B05) [37]. While *SLC29A3* knockdown decreased basal autophagy, *SLC29A1* knockdown led to increased LC3B-II levels and decreased SQSTM1, indicating enhanced autophagy (Figures 1(A,B)).

To validate these opposing effects, we transiently overexpressed *SLC29A1* and *SLC29A3* using N-terminal YFP-tagged constructs. *SLC29A3*-YFP predominantly localized to perinuclear vesicles, consistent with endolysosomal targeting, while *SLC29A1*-YFP largely showed continuous staining at the basolateral plasma membrane (Figure 1C). *SLC29A3-YFP* overexpression increased the proportion of cells exhibiting punctate LC3B-II staining (from 43.8% to 56.2%) and reduced cytosolic LC3B (from 33.8% to 26.1%), indicative of autophagy induction (Figure 1C; Figure S3). In contrast, *SLC29A1-YFP* overexpression reduced the proportion of cells showing LC3B-II puncta (from 43.8% to 29.5%) and increased diffuse LC3B-I staining (from 33.8% to 46.2%), suggesting autophagy suppression (Figure 1C; Figure S3). Western blotting analysis confirmed decreased LC3B-II and increased SQSTM1 levels in *SLC29A1*-YFP expressing cells (Figure 1D). Like these observations in HEK293 cells overexpressing ENTs, primary mouse macrophages with higher *SLC29A3* relative to *SLC29A1* expression exhibited high autophagy, as indicated by enhanced LC3B-II and reduced SQSTM1. In contrast, primary mouse endothelial cells with higher *SLC29A1* than *SLC29A3* expression showed relatively low autophagy, reflected by lesser LC3B-II and higher SQSTM1 accumulation (Figure 1E). Collectively, these findings demonstrate that *SLC29A1* and *SLC29A3* exert opposing effects on basal autophagy: *SLC29A1* attenuates autophagy, while *SLC29A3* enhances it, underscoring the importance of nucleoside transporter localization and function in autophagy regulation.

### Reciprocal regulation of autophagic flux by SLC29A1 and SLC29A3

Monitoring autophagic flux using lysosomal inhibitors is essential for assessing the dynamic progression of autophagy [42,43]. To determine whether *SLC29A1* or *SLC29A3* influences autophagic flux, we analyzed LC3B-II accumulation in the presence or absence of the lysosomal inhibitor chloroquine (CQ). Under these conditions, LC3B-II accumulation reflects autophagosome synthesis independent of lysosomal consumption of autophagic cargo, and the difference in LC3B-II levels indicates the rate of autophagic flux.

**Figure 2.**
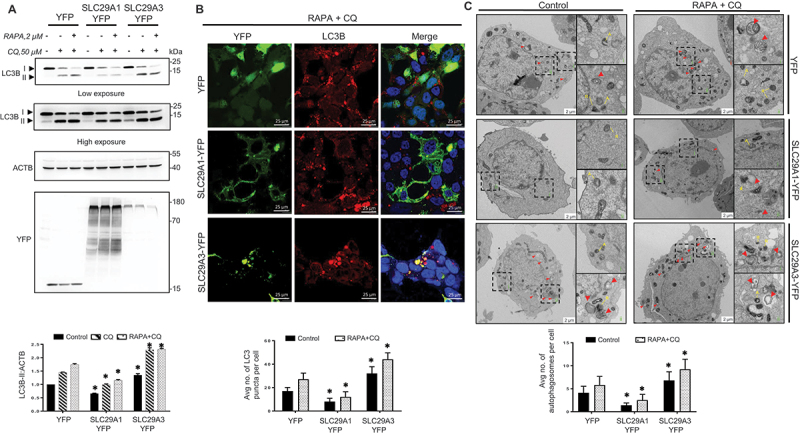
*SLC29A1* suppresses while *SLC29A3* enhances autophagy flux. (A) HEK293 cells transfected with YFP, *SLC29A1*-YFP, or *SLC29A3*-YFP plasmids were treated with 50 μM chloroquine (CQ) with or without 2 μM rapamycin (RAPA) for 4 h to assess autophagy flux. Cell lysates were immunoblotted for LC3B, YFP, and ACTB (loading control). Densitometric quantification of LC3B-II levels normalized to ACTB is shown (*n* = 3). Data are presented as mean ± SEM. *p < 0.05. (B) immunofluorescence analysis of endogenous LC3B (red) in HEK293 cells transfected with YFP, *SLC29A1*-YFP, or *SLC29A3*-YFP and treated with 2 μM RAPA and 50 μM CQ for 4 h. Nuclei were stained with DAPI (blue). Scale bar: 25 μm. LC3B puncta were quantified in 50 cells per condition using ImageJ (*n* = 3). Data are presented as mean ± SEM. *p < 0.05. (C) transmission electron microscopy (TEM) images of HEK293 cells transfected with the indicated constructs. Representative autophagosomes (red, arrowheads) and lysosomes (dense bodies labeled as -A and -R types) are shown (above). Autophagosomes were quantified in 10 cells per condition using ImageJ (*n* = 3). Scale bar: 2 μm. Data are presented as mean ± SEM. *p < 0.05.

In *SLC29A1*-overexpressing cells, CQ treatment resulted in significantly reduced LC3B-II accumulation compared to YFP-transfected controls (Figure 2A), suggesting that *SLC29A1* suppresses autophagy at the initiation stage. In contrast, *SLC29A3*-overexpressing cells exhibited a marked increase in LC3B-II accumulation upon CQ treatment, indicating enhanced autophagic activity (Figure 2A). To assess whether autophagy induction could rescue the suppressed autophagic response in *SLC29A1*-overexpressing cells, we treated cells with rapamycin (RAPA), an MTOR inhibitor known to stimulate autophagy [44], in combination with CQ. In YFP control cells, this combined treatment led to increased LC3B-II levels and decreased LC3-I, confirming enhanced autophagic flux (Figure 2A). *SLC29A3*-overexpressing cells showed a similar robust conversion of LC3B-I to LC3B-II. However, *SLC29A1*-overexpressing cells failed to show any significant increase in LC3B-II or decrease in LC3B-I, even with RAPA treatment, indicating a persistent block in autophagosome initiation (Figure 2A). Supporting this, transcript analysis of *SLC29A1-YFP* versus *SLC29A3-YFP* overexpressing HEK293 cells revealed reduced expression of the autophagy-related gene *ATG5*, a key regulator of autophagosome formation (Fig. S4).

These biochemical findings were corroborated by LC3B immunostaining analysis. *SLC29A1*-YFP cells displayed a significant reduction in LC3B puncta following CQ and RAPA treatment, while *SLC29A3*-YFP cells showed increased puncta intensity and size, consistent with elevated autophagic turnover (Figure 2B). Transmission electron microscopy (TEM) analysis for autophagic vacuoles and lysosomes identified as dense bodies [45] further validated these observations. The dense bodies were either of residual R type with electron opaque contents or A type with demarcated zones of electron lucency. In *SLC29A1*-overexpressing cells, autophagosomes were nearly absent under basal conditions, with only a few residual dense bodies observed (Figure 2C). In contrast, *SLC29A3*-overexpressing cells displayed numerous dense bodies and autophagic vacuoles at various stages of degradation (Figure 2C). Combined CQ and RAPA treatment increased autophagosome and dense body formation in YFP and *SLC29A3*-YFP cells, but far less in *SLC29A1*-YFP cells, reinforcing that *SLC29A1* suppresses autophagosome initiation (Figure 2C). In summary, *SLC29A1* and *SLC29A3* exert opposing effects on autophagic flux in HEK293 cells. While *SLC29A3* promotes autophagy initiation and flux, *SLC29A1* inhibits these processes, highlighting the distinct regulatory roles of cell surface and endolysosomal nucleoside transporters in autophagy.

### SLC29A substrate Ado induces autophagy via PRKAA/AMPK phosphorylation

**Figure 3.**
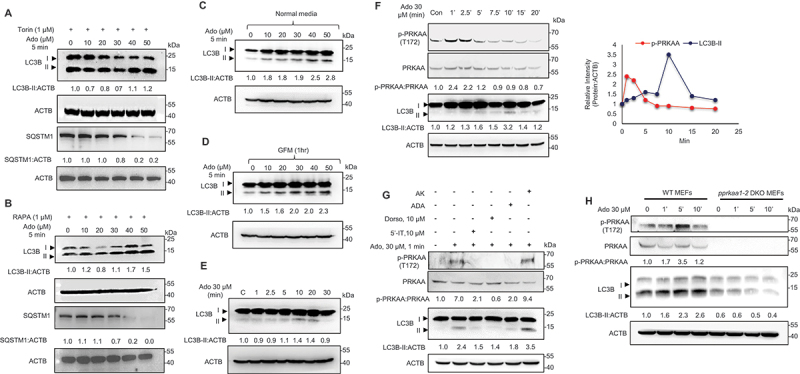
Extracellular Ado induces autophagy in a PRKAA-dependent manner. (A, B) HEK293 cells were treated with autophagy inducers torin1 (1 μM; A) and RAPA (1 μM; B) followed by increasing concentrations of Ado (10–50 μM) for 5 min. Cell lysates were analyzed by Western blotting for LC3B and ACTB (loading control). LC3B-II:ACTB ratios are shown. (C, D) HEK293 cells were treated with increasing concentrations of Ado (10–50 μM) for 10 min under normal (C) or glucose-free medium (GFM) (D) conditions. Cell lysates were analyzed by Western blotting for LC3B and ACTB (loading control). LC3B-II:ACTB ratios are shown (*n* = 3). (E) Western blotting analysis of LC3B levels in HeLa cells treated with 30 μM Ado over a time course. ACTB was used as a loading control. (F) Time-course analysis of HEK293 cells treated with 30 μM Ado for 1–20 min. Lysates were immunoblotted for phospho-PRKAA/AMPK (Thr172), total PRKAA/AMPK, LC3B, and ACTB. Quantification of p-PRKAA:PRKAA and LC3B-II:ACTB ratios is shown (right; *n* = 3). (G) HEK293 cells were treated with 30 μM Ado for 1 or 10 min, with or without 30 min pre-treatment using 5-iodotubercidin (5-IT), dorsomorphin (Dorso), or EHNA, or following transfection with ADA or ADK. Lysates were probed for p-PRKAA/AMPK, PRKAA/AMPK, LC3B, SQSTM1, and ACTB (*n* = 3). (H) wild-type (WT) and *prkaa1 prkaa2* double knockout (dKO) MEFs were treated with 30 μM Ado for 1–10 min. Lysates were analyzed by Western blotting for the indicated proteins. Quantification of p-PRKAA/AMPK:PRKAA/AMPK and LC3B-II:ACTB ratios is shown. Data represent the mean ± SEM (*n* = 3.

Given the central role of kinases such as PRKAA/AMPK, MTOR, and ULK1 (unc-51 like autophagy activating kinase 1) in autophagy initiation, we investigated whether *SLC29A1*-mediated suppression of autophagy involves these signaling pathways. Torin1, like RAPA, is well-known for activating autophagy by inhibiting MTOR signaling and regulating ULK1 [46, 47]. Similarly, glucose deprivation is known to activate autophagy through PRKAA/AMPK, MTOR, and ULK1 signaling, while glucose repletion suppresses autophagy, likely via MTOR activation and PRKAA/AMPK inhibition [48]. Extracellular Ado, a shared substrate of *SLC29A1* and *SLC29A3*, has been reported to induce PRKAA phosphorylation through its conversion to AMP [47]. However, its direct role in autophagy regulation remains less clear. To test whether Ado modulates autophagy via PRKAA/AMPK, we treated HEK293 cells with increasing concentrations of Ado (10–50 μM) for 5 min under RAPA (1 μM), torin1 (1 μM), and glucose-modulated conditions. Optimization experiments with different periods of glucose starvation showed that ~0.5–1 h is sufficient to induce autophagy in this model. Western blotting analysis revealed a dose-dependent increase in LC3B-II levels, which was evident under torin1 and RAPA treated conditions (with decrease in SQSTM1) (Figures 3A, B) as well as glucose starvation for 1 h (Figures 3C, D) indicating enhanced autophagy in Ado treated HEK293 cells. Comparable results were observed in HeLa cells treated with Ado under glucose starvation for 1 h (Figure 3E).

Interestingly, PRKAA/AMPK phosphorylation was not detected within 10 min of Ado treatment [24]. A time-course analysis using 30 μM Ado revealed rapid PRKAA/AMPK phosphorylation beginning at 1 min, peaking at 2.5 min, and declining thereafter (Figure 3F). LC3B-II levels, however, remained elevated up to 10 min of post-treatment (Figure 3F), suggesting that PRKAA/AMPK activation precedes LC3 lipidation. To determine whether Ado’s effects are mediated through its conversion to AMP, we pretreated HEK293 cells with the ADK inhibitor 5’-iodotubercidin. ADK inhibition blocked Ado-induced PRKAA/AMPK phosphorylation and LC3B-II accumulation (Figure 3G). Similarly, pharmacological inhibition of PRKAA/AMPK with dorsomorphin abolished Ado-induced autophagy (Figure 3G). Further, overexpression of ADA (adenosine deaminase), which converts Ado to inosine, suppressed Ado-induced PRKAA/AMPK phosphorylation, while overexpression of ADK (that converts Ado to AMP) enhanced it, confirming that Ado must be metabolized to AMP to activate PRKAA/AMPK and induce autophagy (Figure 3E).

To further confirm the requirement of PRKAA/AMPK in Ado-mediated autophagy, we treated wild-type (WT) and *prkaa1/*AMPKα1 *prkaa2*/AMPKα2 double knockout (dKO) mouse embryonic fibroblasts (MEFs) with Ado. LC3B-II accumulation in the presence of 30 μM Ado was time dependent on WT MEFs (as seen in HeLa and HEK293 cells) with maximal accumulation seen at around 5 min (Figure 3H). Notably, LC3B-II accumulation was observed in WT but not in *prkaa1 prkaa2* dKO MEFs, demonstrating that Ado-induced autophagy is AMPK-dependent (Figure 3H).

### SLC29A1 and SLC29A3 regulate cellular Ado compartmentalization to modulate autophagy

While ENTs are well known to modulate Ado transport across various biological membranes (e.g., plasma membrane, nuclear, mitochondrial, lysosomal), their role in differentially regulating PRKAA/AMPK phosphorylation is not well studied. Hence, the question arose whether the variations in Ado transport and/or cellular compartmentalization of Ado by *SLC29A1* and *SLC29A3* are responsible for the diametrically opposite effects on autophagy. Given the autophagy-inducing effects of Ado [49, 50] and the bidirectional transport properties of *SLC29A1* at the cell surface [51,52], we hypothesize that elevated *SLC29A1* expression may differentially regulate autophagy by modulating Ado influx and efflux, thereby influencing its intracellular retention. Additionally, *SLC29A3* is an acidic-pH dependent transporter that allows unidirectional flux of Ado from the lysosome to the cytosol; therefore, we hypothesized high *SLC29A3* expression may enhance autophagy by facilitating Ado mobilization within intracellular compartments.

**Figure 4.**
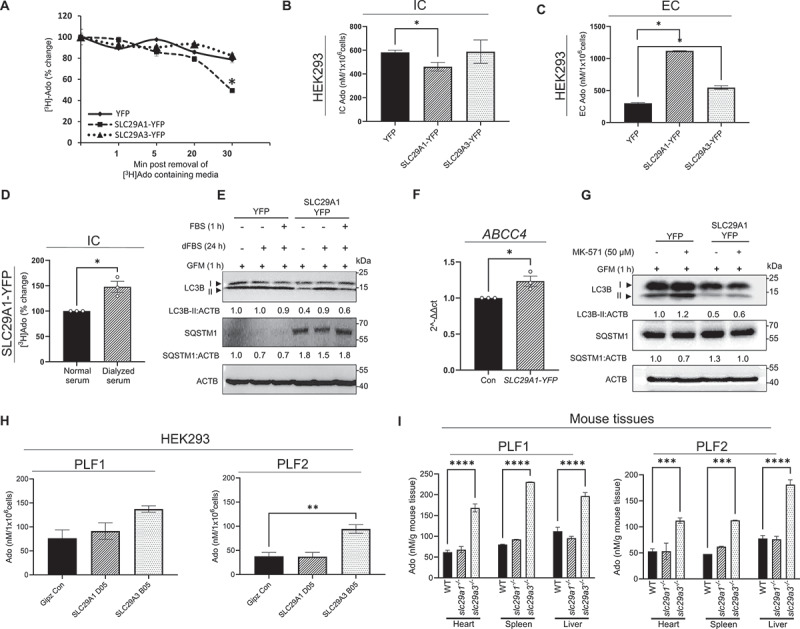
*SLC29A1* and *SLC29A3* impact subcellular compartmentalization of Ado. (A) HEK293 cells transfected with YFP, *SLC29A1*-YFP, or *SLC29A3*-YFP were preloaded with a 10 μM mixture of unlabeled and [^3^ H]Ado for 30 min. After removal of extracellular [^3^ H]Ado, intracellular radioactivity was measured. Data represent percent change in absolute [^3^ H]Ado levels and are presented as mean ± SEM (*n* = 3). *p < 0.05. (B) HEK293 cells transfected with YFP, *SLC29A1*-YFP, and *SLC29A3*-YFP were treated with 30 μM Ado for 30 min, extracellular Ado media removed, and cell lysates analyzed for Ado influx using LC-MS/MS. Bars represent mean ± SEM (*n* = 3). *p < 0.05. (C) In a parallel batch of cells treated with 30 μM Ado for 30 min, media was removed, and cells were washed with ice cold PBS and incubated in 1x sodium buffer at 37°C for 30 min. Supernatant was dried, reconstituted, and analyzed for Ado by LC-MS/MS. Data represent mean ± SEM (*n* = 3). *p < 0.05. (D) Intracellular (IC) [^3^H]Ado levels in HEK293 cells maintained in normal serum vs dialyzed serum at 10% for 24 h. (E) Western blotting analysis of HEK293 cells maintained in normal serum vs dialyzed serum for LC3B, SQSTM1 and ACTB (loading control). LC3B-II:ACTB and SQSTM1:ACTB ratios are shown. (F) qPCR analysis of HEK293 cells expressing YFP and *SLC29A1*-YFP for expression of ABCC4/MRP4. Data is presented as 2^−ΔΔct^. (G) Western blotting analysis of YFP and *SLC29A1*-YFP -expressing HEK293 cells treated with ABCC4/MRP4 inhibitor MK-571 (50 µM) and probed for LC3B, SQSTM1 and ACTB (loading control). LC3-II:ACTB and SQSTM1:ACTB ratios are shown. (H, I) LC-MS/MS analysis of Ado levels in purified lysosomal fractions (PLF) 1 and 2 derived from HEK293 cells expressing *GIPZ*-shRNA (control), *SLC29A1* shRNA, *SLC29A3* shRNA (H) or WT (control), *slc29a1* KO or *slc29a3* KO mouse spleen, cardiac, and hepatic lysates (I) (*n* = 3, mean ± SEM). *p < 0.05, **p < 0.01, ***p < 0.001.

To evaluate these possibilities, we preloaded *YFP*, *SLC29A1* and *SLC29A3* overexpressing HEK293 cells with 10 µM [^3^H]Ado for 30 min. Thereafter, the cells were washed twice with PBS and tracked for intracellular (IC) [^3^H]Ado levels at different time points (1–30 min) after the removal of [^3^H]Ado containing media. The *SLC29A1* overexpressing cells showed a rapid decline in IC [^3^H]Ado concentration over time from between 1 to 30 min after replacement of [^3^H]Ado containing media with Ado-free, Na^+^-buffer (Figure 4A), indicating the decreased retention of radiolabeled Ado within cells in the presence of SLC29A1-YFP. Notably, this rapid decline was not evident in YFP or *SLC29A3*-YFP expressing cells (Figure 4A). Together, these results demonstrate the capability of *SLC29A1* in reducing the concentration of IC Ado and thereby negatively influencing autophagy.

To determine whether the reduced IC Ado levels observed in *SLC29A1*-YFP-expressing cells result from increased Ado efflux, we employed a highly sensitive LC-MS/MS assay (Fig. S5A) to quantify both IC and extracellular (EC) Ado levels in HEK293 cells transiently overexpressing *SLC29A1-YFP* or *SLC29A3-YFP*. This assay, optimized for accurate Ado quantification (Fig. S5B), effectively distinguishes Ado from its immediate metabolic derivatives, including inosine and AMP. Cells expressing YFP alone served as controls. HEK293 cells were preloaded with 30 μM Ado for 30 min (pulse), washed, and then incubated in Ado-free buffer for an additional 30 min (chase). Analysis of the EC buffer revealed a marked increase in Ado levels in *SLC29A1*-YFP-expressing cells with simultaneous reduction in IC Ado content, indicating enhanced Ado efflux as a likely mechanism for the observed reduction in cellular Ado retention (Figure 4B). Although *SLC29A3*-YFP-expressing cells also exhibited a significant increase in EC Ado levels compared to controls, it was only half the amount observed in *SLC29A1*-YFP cells (Figure 4C). The reduction in IC accumulation in *SLC29A1*-YFP cells was also evident when Ado was replaced with 2-chlorodeoxyadenosine, a non-metabolizable analog, reinforcing the role of *SLC29A1* in mediating Ado efflux. Collectively, these findings support the conclusion that *SLC29A1* overexpression reduces IC Ado content through enhanced efflux, thereby contributing to autophagy suppression in HEK293 cells.

Since the predominant export of Ado in HEK293 *SLC29A1*-YFP cells may not fully reflect *in vivo* metabolic states, where concentration gradients and transporter expression levels vary across tissues, we conducted additional experiments under Ado-depleted conditions. To achieve this, we replaced regular fetal bovine serum (FBS) with dialyzed FBS in the complete media that substantially reduces the concentrations of low molecular weight molecules including nucleosides. Interestingly, unlike cells cultured in regular media, which predominantly effluxed [^3^ H]Ado, nucleosides-starved HEK293 *SLC29A1*-YFP cells exhibited a net increase in [^3^ H]Ado uptake (Figure 4D). Interestingly, the enhanced Ado uptake via *SLC29A1* was accompanied by increased conversion of LC3B-I to LC3B-II and reduced SQSTM1 levels indicating autophagy activation, which was abolished upon replenishment with regular FBS (Figure 4E). Taken together, these findings suggest that compared to *SLC29A3*, *SLC29A1* increases EC Ado content by reducing autophagy although a context-dependent role for *SLC29A1* in regulating autophagy cannot be ruled out, primarily influenced by the directionality of Ado flux driven by the cellular metabolic state.

To clarify whether the observed Ado export is mediated exclusively by *SLC29A1* in HEK293 *SLC29A1*-YFP cells and to rule out the potential contribution of active nucleotide exporters such as *ABCC4*/MRP4, we measured the expression of *ABCC4* via qPCR. We found that the ABCC4 transcripts were elevated upon *SLC29A1* overexpression in HEK293 cells under normal growth conditions (Figure 4F). Given that *ABCC4* can export AMP and other nucleotides that are subsequently converted to Ado extracellularly via NT5E/CD73 (5’−nucleotidase ecto), this upregulation may additionally contribute to the increased EC Ado observed with *SLC29A1* overexpression, we examined putative modulation of autophagy upon pharmacological inhibition of *ABCC4* using MK-571. The results showed that MK-571 only modestly mitigated the autophagy-suppressing effects of *SLC29A1*, indicating that *SLC29A1* still plays a major role in the Ado efflux-induced reduction of autophagy in the HEK293 model (Figure 4G).

Although IC Ado levels remained comparable between control (YFP) and *SLC29A3-YFP* overexpressing HEK293 cells, the *SLC29A3* overexpressing cells exhibited a significantly enhanced autophagic response. To investigate whether this discrepancy could be attributed to altered subcellular compartmentalization of Ado, we performed subcellular fractionation in GIPZ control and *SLC29A3* knockdown HEK293 cells to assess Ado distribution in the absence of *SLC29A3*. Given *SLC29A3*’s predominant localization to endolysosomal compartments, we hypothesized that its loss would lead to increased Ado accumulation within lysosomes due to impaired export to the cytosol (Figure 4I). LC-MS/MS analysis of purified lysosomal fractions (PLF1and PLF2) from *SLC29A3* knockdown cells revealed significantly elevated Ado levels compared to controls (Figure 4H), supporting this hypothesis. Similar findings were observed in cardiac, splenic and hepatic lysosomal fractions isolated from WT and *slc29a3* knockout (KO) mice with KO-derived PLFs exhibiting higher accumulation of Ado (Figure 4I). In contrast, no significant differences in lysosomal Ado content were detected in *SLC29A1* knockdown HEK293 or *slc29a1* KO tissue-derived PLFs (Figures 4H, I), indicating that *SLC29A3*, but not *SLC29A1*, regulates lysosomal Ado efflux. Collectively, these results demonstrate that *SLC29A1* reduces cytosolic Ado levels by promoting cellular efflux, thereby suppressing autophagy. In contrast, *SLC29A3* facilitates lysosomal Ado export to cytosol. Loss of *SLC29A3* leads to endolysosomal sequestration of Ado, reducing its cytosolic availability and consequently impairing autophagy activation.

### SLC29A1 and SLC29A3 differentially regulate AMPK phosphorylation

**Figure 5.**
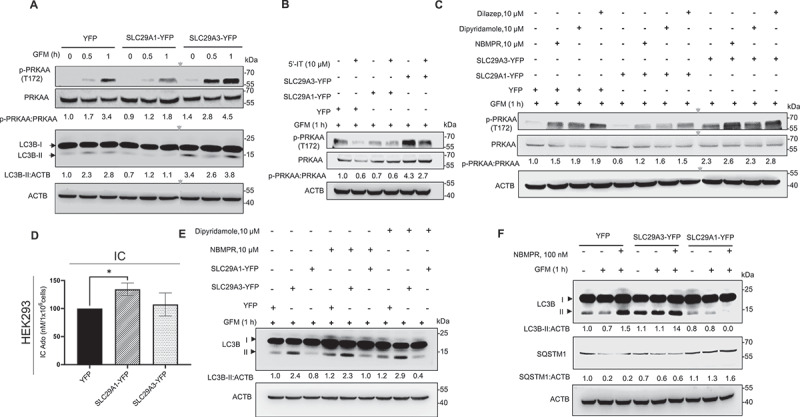
*SLC29A1* and *SLC29A3* differentially regulate AMPK activity. (A) HEK293 cells transfected with YFP, *SLC29A1*-YFP, or *SLC29A3*-YFP were subjected to glucose starvation for 0.5 or 1 h. Lysates were analyzed by immunoblotting for phospho-PRKAA/AMPK (Thr172), total PRKAA/AMPK, and ACTB (loading control). p-PRKAA/AMPK:PRKAA/AMPK ratios were quantified (*n* = 3). *p < 0.05. (B) Cells transfected as above were glucose-starved for 1 h and treated with or without 10 µM 5’-IT (iodotubercidin). Lysates were probed with the indicated antibodies. The p-PRKAA/AMPK:PRKAA/AMPK ratios were calculated. ACTB served as loading control. Data are presented as mean ± SEM (*n* = 3). *p < 0.05. (C) HEK293 cells transfected with the indicated plasmids were glucose-starved for 1 h in the presence or absence of *SLC29A1* inhibitors-NBMPR (10 μM), dipyridamole (10 μM), or dilazep (10 μM). Lysates were analyzed by immunoblotting with the indicated antibodies. ACTB was used as loading control (*n* = 2). *p < 0.05. (D) HEK293 cells were transfected with respective plasmids, treated with 30 μM Ado and 10 μM NBMPR for 30 min. The media was removed, cells were lysed, nucleosides were extracted, dried and analyzed by LC-MS analysis. Data represent mean ± SEM (*n* = 2). *p < 0.05. (E) western blotting analysis of LC3B in HEK293 cells transfected with YFP, *SLC29A3*-YFP, or *SLC29A1*-YFP and treated with 10 μM NBMPR or 10 μM dipyridamole under glucose starvation. (F) Western blotting analysis of LC3B and SQSTM1 in HEK293 cells transfected with the same constructs and treated with 100 nM NBMPR under normal and glucose-starved conditions. ACTB was used as a loading control. Arrowheads in the figure point at non-contiguous lanes.

Given the distinct effects of *SLC29A1* and *SLC29A3* on subcellular Ado compartmentalization, we next investigated whether these transporters differentially regulate PRKAA/AMPK phosphorylation. HEK293 cells expressing YFP, *SLC29A1*-YFP, or *SLC29A3*-YFP were subjected to glucose starvation for 0.5 h and 1 h followed by Western blotting analysis of total cell lysates. *SLC29A3* overexpression significantly enhanced PRKAA/AMPK phosphorylation under both basal and glucose-deprived conditions (clearer at 1 h glucose starvation), whereas *SLC29A1* overexpression led to a greater than 50% reduction in PRKAA/AMPK phosphorylation (Figure 5A). Consistent with these findings, LC3B-II levels were elevated in *SLC29A3* overexpressing cells and reduced in *SLC29A1* overexpressing cells (Figure 5A).

To determine whether Ado’s effects on PRKAA/AMPK activation are mediated through its conversion to AMP, cells were treated with the ADK inhibitor 5′-iodotubercidin. This treatment markedly reduced PRKAA/AMPK phosphorylation across all three cell types (YFP, *SLC29A1*-YFP, and *SLC29A3*-YFP), confirming that Ado must be phosphorylated to AMP to activate PRKAA/AMPK (Figure 5B). Furthermore, overexpression of ADK enhanced Ado-induced PRKAA/AMPK phosphorylation, while overexpression of ADA suppressed it, further supporting the role of AMP as the key mediator in this signaling axis (Figure 3G).

Furthermore, we tested the effects of pharmacological *SLC29A1* transport inhibitors – NBMPR, dipyridamole, and dilazep [53] on PRKAA/AMPK activation. Inhibition of *SLC29A1* transport by all three inhibitors increased PRKAA/AMPK phosphorylation in control and *SLC29A1* overexpressing cells and further enhanced PRKAA/AMPK activation in *SLC29A3* overexpressing cells (Figure 5C) perhaps due to a combined effect of *SLC29A3* mobilization of Ado to cytosol from the lysosomes and reduction in Ado efflux (due to inhibition of endogenous SLC29A1). Corroboratively, treatment with the NBMPR significantly increased IC Ado levels in *SLC29A1* overexpressing cells, as quantified by LC-MS/MS analysis (Figure 5D). Further, we examined the role of pharmacological *SLC29A1* inhibitors on LC3B-I to LC3-II conversion in *SLC29A1*-YFP and *SLC29A3*-YFP cells. Both NBMPR and dipyridamole increased LC3B-I to LC3B-II conversions in both cell types; *SLC29A3*-YFP cells much more prominently than in *SLC29A1*-YFP cells (Figure 5E). Furthermore, treatment with NBMPR not only increased LC3-I to LC3B-II conversion but also reduced SQSTM1 levels in *SLC29A3*-YFP cells but not in *SLC29A1*-YFP cells (Figure 5F). Thus, these results confirmed *SLC29A1*’s effect on autophagy is Ado transport-dependent, and pharmacological inhibition of *SLC29A1* transport is sufficient to increase cytosolic retention of Ado to induce autophagy in HEK293 cells. Moreover, these results underscore the critical role of *SLC29A1*-mediated Ado efflux in suppressing PRKAA/AMPK activation and autophagy, while *SLC29A3* promotes autophagy by increasing Ado movement within IC compartments.

### SLC29A1 and SLC29A3 differentially regulate the PRKAA-MTOR-ULK1 pathway and BECN1-BCL2 interaction to modulate autophagy

**Figure 6.**
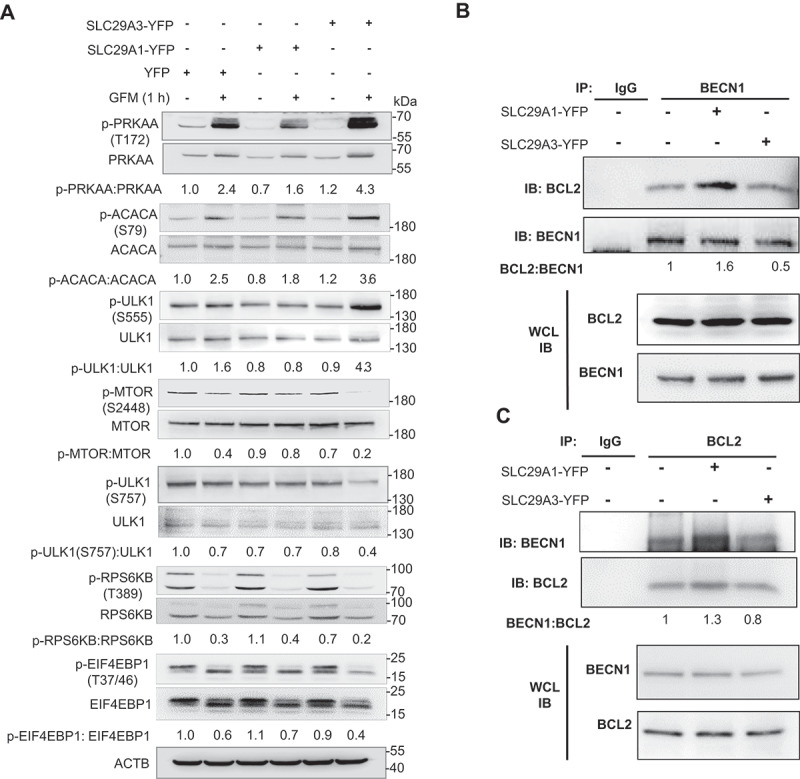
*SLC29A1* and *SLC29A3* differentially modulate the PRKAA-MTOR-ULK1 signaling axis and BECN1-BCL2 interaction. (A) HEK293 cells were transfected with YFP, *SLC29A1*-YFP, or *SLC29A3*-YFP for 24 h, followed by 1 h of glucose starvation. Cell lysates were analyzed by immunoblotting for phospho-PRKAA/AMPK (Thr172), total PRKAA/AMPK, phospho-ACACA (Ser79), total ACACA, phospho-ULK1 (Ser555 and Ser757), total ULK1, phospho-MTOR (Ser2448), total MTOR, phospho-RPS6KB (Thr389), total RPS6KB, phospho-EIF4EBP1 (Thr37/46), and total EIF4EBP1. ACTB was used as a loading control (*n* = 3). (B–C) HEK293 cells transfected with the indicated plasmids were subjected to co-immunoprecipitation (co-IP) assays. (B) Cell lysates were immunoprecipitated with anti-BECN1 antibody or rabbit monoclonal IgG isotype control, followed by immunoblotting for BCL2. (C) Reciprocal co-IP was performed using anti-BCL2 antibody, followed by immunoblotting for BECN1. Whole cell lysates (WCL) were also probed with the indicated antibodies (*n* = 3).

PRKAA/AMPK is a central energy sensor that regulates autophagy by phosphorylating ULK1 at Ser555 and inhibiting MTOR activity [54, 55]. To elucidate the downstream signaling events influenced by *SLC29A1* and *SLC29A3*, we analyzed the phosphorylation status of PRKAA/AMPK and its downstream targets – phospho-ACACA and phospho-ULK1 (Ser555). As expected, *SLC29A3* overexpression enhanced glucose starvation-induced phosphorylation of PRKAA/AMPK, ACACA, and ULK1 (Ser555), consistent with its role in promoting autophagy (Figure 6A). In contrast, *SLC29A1* overexpression suppressed PRKAA/AMPK and ULK1 (Ser555) phosphorylation under glucose starvation, without significantly affecting ACACA phosphorylation (Figure 6A). This suggests that *SLC29A1* selectively impairs PRKAA/AMPK activation. Further analysis of the MTOR pathway revealed that *SLC29A3* significantly inhibited phosphorylation of MTOR and its downstream targets – ULK1 (Ser757), RPS6KB/S6K, and EIF4EBP1/4E-BP1 under glucose-starved conditions (Figure 6A). *SLC29A1*, however, did not alter MTOR or its downstream signaling (Figure 6A), indicating that its inhibitory effect on autophagy is independent of MTOR regulation.

To explore alternative mechanisms, we examined the interaction between BECN1 and BCL2, a known inhibitory complex that suppresses autophagy by preventing BECN1’s association with class III phosphatidylinositol 3-kinase [56, 57]. Since *SLC29A1* negatively affects autophagy flux and it did not influence the MTOR signaling, we hypothesized that *SLC29A1* may instead influence the binding of BCL2 to BECN1 to modulate autophagy. Co-immunoprecipitation (co-IP) assays in HEK293 cells revealed that *SLC29A1* overexpression enhanced BECN1-BCL2 binding, despite no changes in their total protein levels (Figure 6B). This was confirmed by reciprocal co-IP experiments, further supporting the notion that *SLC29A1* suppresses autophagy by stabilizing the BECN1-BCL2 complex (Figure 6C). In contrast, *SLC29A3* overexpression did not significantly affect BECN1-BCL2 interaction (Figures 6B, C), reinforcing that *SLC29A3* promotes autophagy primarily through PRKAA/AMPK activation and MTOR inhibition, whereas *SLC29A1* suppresses autophagy via reduced PRKAA/AMPK signaling and enhanced BECN1-BCL2 binding. Together, these findings highlight distinct mechanistic pathways through which *SLC29A1* and *SLC29A3* regulate autophagy: *SLC29A3* activates the PRKAA/AMPK-MTOR-ULK1 axis to promote autophagy, while *SLC29A1* inhibits autophagy by dampening PRKAA/AMPK signaling and reinforcing BECN1-BCL2 interaction.

### *In vivo* organ analysis reveals enhanced and impaired autophagy in slc29a1^−/−^ and slc29a3^−/−^ mice, respectively

**Figure 7.**
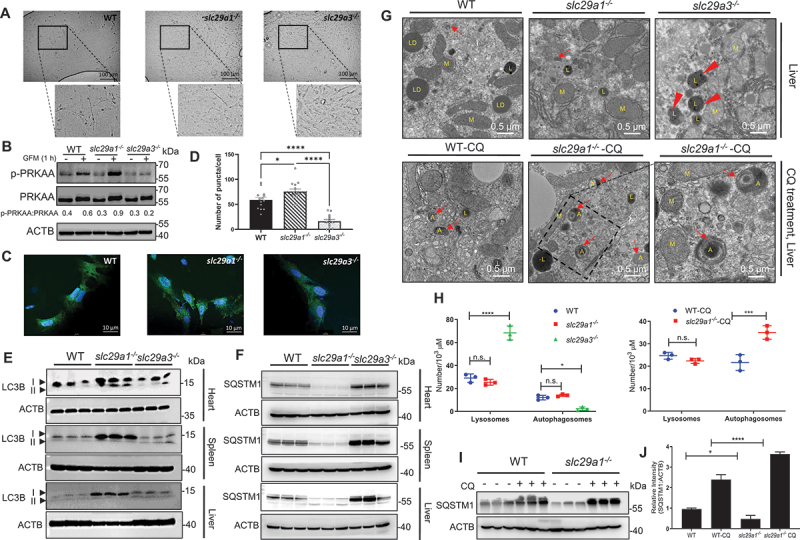
*In vivo* analysis of *slc29a1*^−/−^ and *slc29a3*^−/−^ mice. (A) Representative brightfield microscopy images showing wild-type (WT), *slc29a1*^−/−^ and *slc29a3*^−/−^ mouse embryo fibroblasts (MEFs). Scale bars: 100 μm. (B) lysates of WT, *slc29a1*^−/−^ and *slc29a3*^−/−^ MEFs were analyzed by immunoblotting with the indicated antibodies. ACTB was used as a loading control. (C) immunofluorescence analysis of endogenous LC3B (green) in WT, *slc29a1*^−/−^ and *slc29a3*^−/−^ MEFs. Nuclei were stained with DAPI (blue). Scale bar: 10 μm. (D) LC3B puncta were quantified and plotted. Data are presented as mean ± SEM. *p < 0.05, ****p ≤ 0.0001. (E & F) tissue lysates from heart, spleen, and liver of WT, *slc29a1*^−/−^ and *slc29a3*^−/−^ mice were analyzed by immunoblotting with LC3B (E) and SQSTM1 (F) antibodies. ACTB was used as a loading control. (G) Representative transmission electron micrographs of liver tissues from WT, *slc29a1*^−/−^ and *slc29a3*^−/−^ mice. Arrows indicate autophagosomes and lysosomes. Scale bars: 0.5 μm and 0.1 μm. (H) Quantification of lysosomes and autophagosomes was performed in 10 fields per sample using ImageJ. Data represent mean ± SEM from two independent experiments. *p < 0.05, ***p ≤ 0.001, ****p ≤ 0.0001. (I) liver lysates from WT and *slc29a1*^−/−^ mice treated with saline or chloroquine were analyzed by Western blotting for autophagy markers. (J) Relative intensity of SQSTM1 was quantified. Data represent mean ± SEM from three independent experiments. *p < 0.05, ***p ≤ 0.001, ****p ≤ 0.0001.

To investigate the physiological relevance of *SLC29A1* and *SLC29A3* in autophagy regulation, we analyzed autophagic activity in WT, *slc29a1*^*-/-*^, and *slc29a3*^−/−^ mice. As previously reported, *slc29a3*^*-/-*^ mice exhibited lysosomal dyshomeostasis, anemia and compromised mesenchymal tissue integrity with reduced lifespan [37, 58], whereas *slc29a1*^*-/-*^ mice develop skeletal abnormalities such as aberrant bone density, dorsal spine curvature, and compromised locomotor coordination at later stages of life (>10 months) [59,60]. To explore the role of ENTs in Ado-mediated PRKAA/AMPK signaling during development, we isolated and examined MEFs derived from *slc29a1*^*-/-*^ and *slc29a3*^*-/-*^ mice in comparison with MEFs derived from WT mice (Figure 7A). The MEFs isolated from both groups of mice (*slc29a1*^*-/-*^ and *slc29a3*^*-/-*^) did not exhibit any overt morphological differences in comparison to WT MEFs (Figure 7A). However, MEFs isolated from *slc29a1*^*-/-*^ mice exhibited increased PRKAA/AMPK phosphorylation relative to MEFs isolated from WT mice indicating enhanced autophagy (Figure 7B). In contrast, *slc29a3*^*-/-*^ MEFs displayed reduced PRKAA/AMPK phosphorylation (Figure 7B). Confocal imaging showed an increased LC3B puncta in *slc29a1*^−/−^ MEFs compared to WT MEFs and *slc29a3*^*-/-*^ MEFs (Figures 7C, D) suggesting ENTs indeed differentially modulate autophagy by altering PRKAA/AMPK phosphorylation *in vivo*.

To assess autophagy in mature mouse tissues, we collected heart, spleen, and liver tissues from 12-week-old mice (*n* = 3 per genotype) and analyzed LC3B and SQSTM1 levels by Western blotting. *slc29a1*^*-/-*^ tissues exhibited significantly higher LC3B and reduced SQSTM1 accumulation, indicative of enhanced autophagic flux, whereas *slc29a3*^*-/-*^ tissues showed lower or comparable LC3B levels as WT tissues but with marked SQSTM1 accumulation, consistent with impaired autophagy (Figures 7(E, F); Figure S6). No sex-specific differences were observed with any of these markers. TEM analysis of liver tissues further supported these findings. Under basal conditions, *slc29a1*^*-/-*^ livers did not show increased autophagosome numbers (Figures 7(G, H)). However, following CQ treatment (50 mg/kg, 4 h), *slc29a1*^*-/-*^ livers displayed a pronounced accumulation of double-membrane autophagosomes compared to WT, indicating robust autophagic flux (Figures 7(G, H)). In contrast, *slc29a3*^*-/-*^ livers exhibited fewer autophagosomes and enlarged lysosomes containing undigested material, even without CQ treatment, suggesting a block in basal autophagic degradation (Figures 7(G, H)). Western blotting analysis of liver lysates from saline- and CQ-treated mice confirmed these observations. CQ treatment led to increased SQSTM1 accumulation in *slc29a1*^*-/-*^ livers, further supporting high autophagic turnover (Figures 7(I, J)).

### Autophagy perturbation reciprocally regulates SLC29A1 and SLC29A3 expression

**Figure 8.**
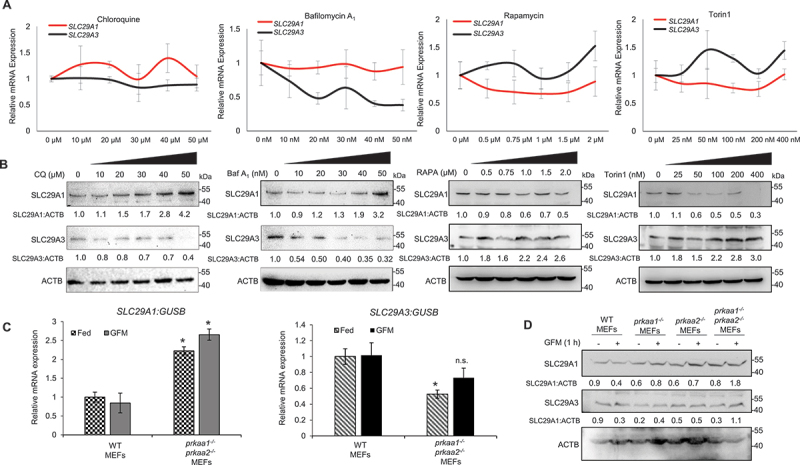
Autophagy oppositely regulates the expression of *SLC29A1* and *SLC29A3*. (A) HEK293 cells were treated with increasing concentrations of autophagy inhibitors (chloroquine [CQ], bafilomycin A_1_ [baf A_1_]) or autophagy inducers (rapamycin [RAPA], torin1). After the treatment, qPCR analysis was performed to analyze the expression of *SLC29A1* and *SLC29A3* transcripts. Human *GUSB* expression was used as an internal control. Data are presented as mean ± SEM from three independent experiments. *p < 0.05. (B) Lysates were subjected to western blotting for *SLC29A1* and *SLC29A3* protein expression with ACTB used as a loading control. (C) Wild-type (WT), *prkaa1* knockout, *prkaa2* knockout, and *prkaa1prkaa2* double knockout MEFs were cultured in either normal or glucose-free media. *SLC29A1* and *SLC29A3* mRNA expression levels were measured by qPCR. Data are presented as mean ± SEM from three independent experiments. *p < 0.05. (D) Lysates were also subjected to Western blotting analysis for mouse *SLC29A1* and *SLC29A3* protein expression with ACTB used as a loading control.

Given the opposing roles of *SLC29A1* and *SLC29A3* in autophagy regulation, we investigated whether autophagy itself could reciprocally influence the expression of these two nucleoside transporters [61, 62]. To explore this further, HEK293 cells were treated with autophagy inhibitors (CQ and bafilomycin A_1_) and inducers (RAPA and torin1). Autophagy inhibition led to a dose-dependent increase in endogenous *SLC29A1* transcript and protein expression, while *SLC29A3* transcript and protein levels decreased steadily (Figures 8(A, B)). Conversely, autophagy induction resulted in a dose-dependent decrease in *SLC29A1* expression and a corresponding increase in *SLC29A3* expression (Figures 8(A, B)). These findings suggest a possible correlation between autophagy activity and ENT expression. To validate these observations in a genetic model of autophagy deficiency, we analyzed *slc29a1* and *slc29a3* expressions in WT and *prkaa1 prkaa2* dKO MEFs. *prkaa1 prkaa2* dKO MEFs exhibited a two-fold increase in *slc29a1* mRNA and a two-fold decrease in *slc29a3* mRNA under both basal and glucose-starved conditions (Figure 8C), further supporting the notion that autophagy deficiency upregulates *SLC29A1* and downregulates *SLC29A3*. Consistent with these findings, Western blotting analysis revealed a corresponding increase in *slc29a1* protein levels and a decrease in *slc29a3* protein levels in *prkaa1 prkaa2* KO MEFs (Figure 8D).

**Figure 9.**
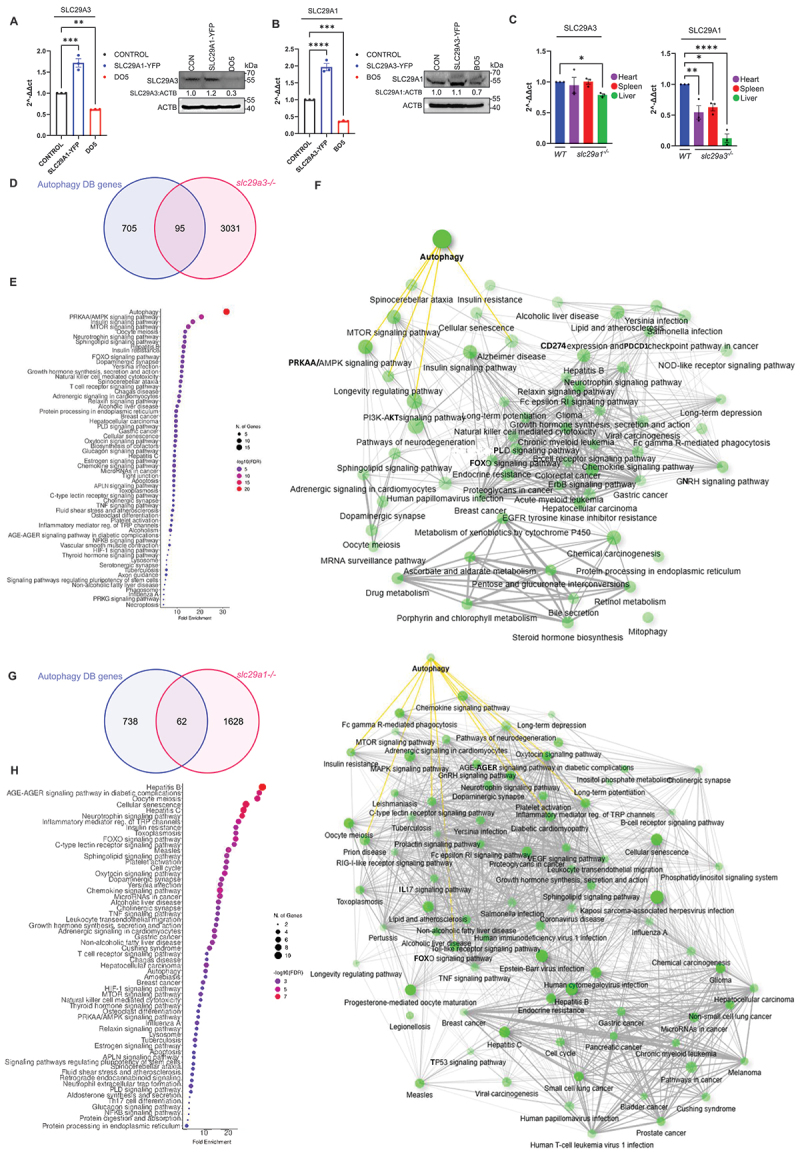
Compensatory actions of *SLC29A* in autophagy. (A, B) HEK293 cells transfected with *SLC29A1*-YFP and *SLC29A3*-YFP for 24 h for overexpression and stably expressing *SLC29A1* (D05) or *SLC29A3* (B05) shRNA for knockdown were subjected to qPCR and immunoblotting analyses for *SLC29A3* and *SLC29A1* gene expression. qPCR data are presented as 2^–ΔΔCt^ relative to control. *p < 0.05. ACTB was used as a loading control for immunoblotting experiments. (C) qPCR analysis of tissues (heart, spleen and liver) isolated from *slc29a1*^−/−^ mice and *slc29a3*^−/−^ mice for *slc29a3* and *slc29a1* transcripts, respectively. qPCR data are presented as 2^–ΔΔCt^ relative to control. *p < 0.05. (D, G) Venn diagram depicting autophagy-regulating genes overlapping between autophagy gene dataset in autophagy database vs DEGs in *slc29a1*^−/−^ and *slc29a3*^−/−^ mice tissues. (E, H) Dot plot of top 60 KEGG pathways enriched in microarray analysis of *slc29a3*^−/−^ mice (spinal tissue) and *slc29a1*^−/−^ (liver tissue) sorted by Fold enrichment with FDR cutoff of 0.5. (F, I) KEGG network analysis of top 60 pathways enriched in *slc29a1*^−/−^ (spinal tissue) and *slc29a3*^−/−^ (liver tissue) sorted based on FDR cutoff of 0.5 and edge cutoff of 0.3. Pathway plots are generated using ShinyGO 0.77.

We next examined whether *SLC29A1* and *SLC29A3* expression levels are subject to compensatory regulation. Quantitative PCR and Western blotting analysis in HEK293 cells stably silenced for *SLC29A1* or *SLC29A3* revealed a significant decrease in *SLC29A3* or *SLC29A1* expression, respectively, suggesting a feedback mechanism aimed at balancing autophagy levels (Figures 9A, B). Conversely, overexpression of *SLC29A1* or *SLC29A3* led to increased expression of the reciprocal transporter, further supporting a compensatory regulatory loop (Figures 9A, B). Despite partial compensation of individual ENTs in the HEK293 model, our findings revealed distinct functional differences in autophagic regulation mediated by *SLC29A1* and *SLC29A3*, perhaps due to the pronounced ectopic overexpression of these two transporters. To further investigate the reciprocal regulation of *SLC29A1* and *SLC29A3* expression, we analyzed their expression profiles across multiple tissues – spleen, liver, and heart – in individual knockout mice (*slc29a1*^*-/-*^ and *slc29a3*^*-/-*^). These data reveal a degree of coregulation existing between *slc29a1* and *slc29a3*, suggesting a partial compensatory mechanism among these ENTs may contribute to organismal survival (Figure 9C). Moreover, to examine additional genes or pathways differentially regulated by *SLC29A1* or *SLC29A3*, we performed microarray analysis on liver tissue from *slc29a3*^*-/-*^ mice. This analysis showed 3031 genes exhibiting differential expression between WT and *slc29a3*^*-/-*^ mice (based on ≥2-fold change; *p* ≤ 0.05). Cross-referencing *SLC29A3*-regulated genes with known autophagy-related genes in the Autophagy Database [63] revealed a distinct subset of genes that was significantly upregulated or downregulated in *slc29a3*^*-/-*^ mice (Figure 9D; Table S1). The Kyoto Encyclopedia of Genes and Genomes (KEGG) network analysis of DEGs in *slc29a3*^*-/-*^ mouse hepatic tissue identified prominent alterations in the autophagy and PRKAA/AMPK signaling pathways (Figures 9(E, F)). In contrast, bioinformatic analysis of existing microarray data from *slc29a1*^*-/-*^ mouse spinal tissue [64] showed relatively fewer changes in autophagy-related genes (Figure 9G; Table S2) perhaps attributable to *SLC29A1*’s context-dependent role in autophagy regulation and the milder phenotypes observed in *slc29a1*^*-/-*^ mice. This was also seen in the KEGG network analysis of DEGs from *slc29a1* null spinal tissue where less enrichment with moderate fold enrichment of autophagy and PRKAA/AMPK pathway genes were observed (Figures 9(H,I)). Furthermore, comparative analysis of *slc29a1*^−/−^ and *slc29a3*^*-/-*^ tissue gene sets revealed shared genes (upregulation of *Stk10*, *Prkcd*, *Rel* and *Lyn*; downregulation of *Atg4a*) that may be involved in compensatory regulation of autophagy (Tables S1-S2). Together, these findings reveal a potential dynamic interplay between autophagy and ENTs expression. *SLC29A1* and *SLC29A3* not only regulate autophagy but are themselves regulated by autophagic activity, forming a reciprocal feedback mechanism that may help maintain autophagic homeostasis.

## Discussion

**Figure 10.**
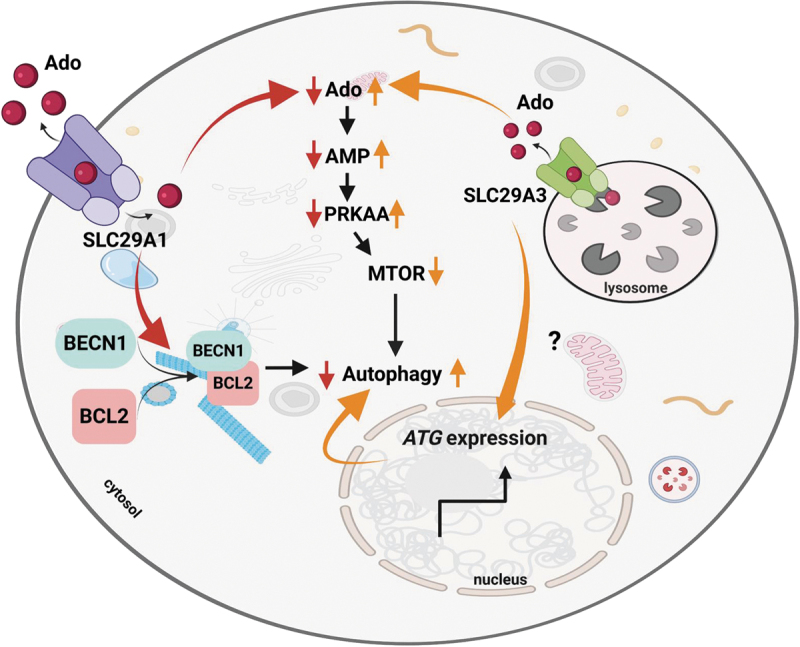
Schematic representation of *SLC29A*’s involvement in the regulation of autophagy. *SLC29A1* and *SLC29A3* differentially regulate autophagy through distinct mechanisms. High *SLC29A1* expression facilitates Ado efflux, reducing IC Ado and AMP levels, which leads to decreased PRKAA/AMPK phosphorylation and suppression of autophagy initiation. *SLC29A1* also enhances BECN1-BCL2 binding, further inhibiting autophagy. In contrast, *SLC29A3* promotes lysosomal Ado efflux into the cytosol, increasing intracellular Ado and AMP concentrations, thereby activating PRKAA, inhibiting MTOR, and enhancing autophagy. *SLC29A3* also upregulates autophagy related (*ATG*) gene expression through currently unknown pathways. Large, curved arrows indicate the signaling pathways and molecular players influenced by *SLC29A1* (red) and *SLC29A3* (orange), while small arrows represent the direction of change associated with each transporter.

This study uncovers crucial roles for nucleoside transporters *SLC29A1* and *SLC29A3* in modulating autophagy through differential regulation of Ado (sub)cellular transport and compartmentalization as well as associated signaling pathways. *SLC29A3* promotes autophagy by activating the PRKAA/AMPK-MTOR axis, whereas *SLC29A1* suppresses autophagy by reducing PRKAA/AMPK phosphorylation although this effect was dependent on the directionality of Ado flux driven by the metabolic state of cells. The effect of *SLC29A1* on autophagy is dependent on its Ado transport function, as pharmacological inhibition of *SLC29A1* is sufficient to increase both PRKAA/AMPK phosphorylation and autophagic activity because of increased IC accumulation of Ado. Mechanistically, *SLC29A1* allows the efflux of IC Ado, limiting its conversion to AMP and subsequent PRKAA/AMPK activation. In contrast, *SLC29A3* mediates lysosomal Ado mobilization into the cytosol, enhancing AMP formation and PRKAA/AMPK activation (Figure 10). Consistent with these roles, *SLC29A1*-deficient cells and tissues exhibit elevated autophagic flux, while *SLC29A3* deficiency impairs autophagy and exhibits lysosomal abnormalities. Further, *SLC29A1* expression enhances BECN1-BCL2 interaction, sequestering BECN1 and inhibiting autophagy. Collectively, these findings highlight the opposing roles of *SLC29A1* and *SLC29A3* in autophagy regulation, driven by differences in Ado transporter cellular localization and resultant Ado compartmentalization (Figure 10).

Ado is a signaling nucleoside that activates cognate trimeric G protein coupled receptors (e.g., adenosinergic receptor signaling) in response to cellular stress [65]. The translocation of Ado from the EC milieu to cytosol by membrane-bound transporters is reported to terminate Ado receptor signaling through ligand depletion [66]. *SLC29A1* exhibits unique transport characteristics compared to concentrative nucleoside transporters (CNTs) such as *SLC28A2*, which mediate unidirectional, Na^+^-coupled Ado influx. In contrast, *SLC29A1* functions as a bidirectional, facilitative transporter, enabling both influx and efflux of Ado based on concentration gradients. This bidirectionality allows *SLC29A1* to dynamically regulate IC Ado levels in response to metabolic demands and EC conditions, as also noted in this study. Unlike CNTs, *SLC29A1*’s passive transport mechanism also enables it to respond to rapid shifts in Ado gradients, which are influenced by metabolic conversion of Ado to AMP (via AK) or inosine (via ADA) intracellularly, and by EC ectonucleotidase activity that increases Ado levels upon breakdown of nucleotides. Interestingly, *SLC29A1*-mediated Ado efflux reduces or keeps AMP formation and PRKAA/AMPK phosphorylation in check, thereby suppressing autophagy under normal growth conditions. However, it should be noted that *SLC29A1* is also capable of increasing cellular influx of Ado under certain nutrient-depleted, metabolic conditions that can promote autophagy. These findings highlight *SLC29A1*’s differential role in modulating cellular energy sensing and autophagic flux. Nevertheless, *SLC29A1*’s action on Ado mobilization from the cytosol to EC space can be distinctly different from the actions of another member within the same ENT family (i.e., *SLC29A3*), which predominantly resides in lysosome and conducts an acidic-pH dependent lysosomal efflux of Ado to cytosol [30,32,52,67]. Importantly, such variations in the directionality of Ado transport and subcellular compartmentalization of Ado play markedly distinct roles in the formation of AMP that triggers Thr172 phosphorylation of PRKAA/AMPK and cellular modulation of autophagic flux.

Because numerous disease states show alterations in the expression of *SLC29A1* [34,35,68], our findings on *SLC29A1* present relevance to pathophysiological conditions as well. Ado receptor ADORA2B/A2b is also reported to contribute to ENT-dependent protective effects in liver injury. ENT expression is also responsive to inflammatory cytokines such as IL1A/IL1, IL12A/IL12, TNF/TNF-α, IFNG/IFN-γ, and CSF2/GM-CSF. For example, *SLC29A3* is induced by IFNB/IFN-β via STAT1 signaling, potentially enhancing autophagy as a host defense mechanism [64, 69]. Pathogens such as *Staphylococcus aureus* and *Pseudomonas aeruginosa* exploit or modulate ENT expression [70], with *SLC29A1* inhibition shown to mitigate infection-induced tissue damage [71]. Beyond infection, Ado and *SLC29A1* play critical roles in cardiovascular, pulmonary, and hepatic protection [72–74]. *SLC29A1* inhibition or deletion has been shown to reduce myocardial infarction and protect against ischemia-reperfusion injury [41]. Pharmacological *SLC29A1* inhibitors like NBMPR, dipyridamole, and dilazep, clinically used for cardiovascular and renal conditions, enhance tissue Ado levels and may promote autophagy, contributing to their therapeutic effects. These findings support that ENTs differentially regulate Ado compartmentalization and autophagy, with implications for diseases involving ischemia, infection, and inflammation. Interestingly, autophagy too plays an important cardioprotective and hepatoprotective roles [71–73]. *SLC29A1* inhibition may confer protection by elevating IC Ado and enhancing autophagic responses, aligning with the observed benefits in models of myocardial infarction and acute lung injury [23,34,35,74]. The dynamic interplay of *SLC29A1* and *SLC29A3* in controlling cellular compartmentalization of Ado to modulate autophagy (the current findings) and the changes in the expression of these ENTs in myocardial infarcts, ischemic tissues, and during tissue damage [23,34,35,74–76] lends further credence to this assertion. In this regard, *SLC29A2*/ENT2, another cell surface-localized ENT, has previously been implicated in autophagy regulation through its role in maintaining the IC balance of nucleosides and nucleobases. Disruption of *SLC29A2* function can lead to the accumulation of modified nucleosides, thereby triggering an autophagic response as well [77]. Therefore, our findings are consistent with the involvement of ENTs in exerting the beneficial actions of endogenous Ado during cellular insults and tissue damage.

Our findings demonstrate that Ado induces PRKAA/AMPK phosphorylation in HEK293 and other cells in a dose-dependent manner, which subsequently leads to increased LC3B-II lipidation, a hallmark of autophagy. Notably, PRKAA/AMPK phosphorylation precedes LC3B-II accumulation, indicating that PRKAA/AMPK activation is an upstream event in autophagy initiation, consistent with previous reports [78]. The autophagic effects of Ado are AMPK-dependent, as Ado treatment fails to induce LC3B-II in *prkaa1 prkaa2* dKO MEFs. Furthermore, IC modulation of Ado metabolism via inhibition of ADK or ADA reduces PRKAA/AMPK phosphorylation, underscoring the importance of intracellular Ado conversion in PRKAA/AMPK activation. PRKAA/AMPK activation at Thr172 is known to promote autophagy by phosphorylating ULK1 at Ser317 and Ser777 [52,79], or by disrupting BECN1-BCL2 interactions. In our study, *SLC29A1* expression suppressed PRKAA/AMPK activity, as evidenced by reduced phosphorylation of ACACA (a downstream PRKAA/AMPK target) and ULK1 at Ser555 under glucose starvation. In contrast, *SLC29A3* expression significantly enhanced these phosphorylation events, indicating opposing roles of *SLC29A1* and *SLC29A3* in PRKAA/AMPK regulation. While SLC29A3 expression modulated MTOR signaling under glucose starvation, *SLC29A1* did not significantly affect MTOR activity. Instead, *SLC29A1* enhanced BECN1-BCL2 binding, further contributing to autophagy inhibition. *SLC29A3* had no such effect, reinforcing the mechanistic divergence between these transporters.

The *slc29a1*^*-/-*^ mice, originally developed to study ethanol pharmacodynamics, exhibit a range of phenotypes including cardioprotection, increased ethanol consumption, reduced body weight, and age-related bone abnormalities [59,60,80–82]. In this study, *slc29a1*^*− /−*^ tissues displayed elevated autophagy, marked by reduced SQSTM1 levels, which may at least partially contribute to the observed cardioprotective phenotype. This aligns with previous findings where autophagy induction (e.g., via *ATG7* expression) mitigates cardiac hypertrophy and enhances survival [83]. However, excessive autophagy has detrimental effects on bone health [84,85], consistent with the ectopic spinal mineralization observed in aged *slc29a1*^*− /−*^ mice, resembling diffuse idiopathic skeletal hyperostosis in humans. *SLC29A1* inhibition has shown therapeutic potential in cerebral ischemia, liver ischemia, and myocardial infarction [86–88], likely through increased IC Ado and autophagy induction. In contrast, *SLC29A3* deficiency results in severe autophagic impairment, hematopoietic failure, and early mortality, reflecting its critical role in adult stem cell maintenance and tissue integrity. These phenotypes mirror human *SLC29A3*-related disorders (e.g., H syndrome, pigmented hypertrichotic dermatosis with insulin-dependent diabetes/PHID, Rosai-Dorfman-Destombes disease/RDD, sinus histiocytosis with massive lymphadenopathy/SHML, dysosteosclerosis) [37,89].

While *SLC29A1* and *SLC29A3* both regulate Ado compartmentalization and autophagy, *SLC29A3* appears to exert a more potent and essential role as the *slc29a3*^*-/-*^ mice succumb to severe phenotypes and early mortalities (~20 weeks). The milder phenotypes in *slc29a1*^*− /−*^ mice suggest possible compensatory mechanisms that may buffer autophagic flux changes in the absence of *slc29a1*. The expression levels of *SLC29A1* and the cell’s metabolic state would also be likely to influence the directionality of Ado flux and magnitude of autophagy response. Autophagy is a tightly regulated, essential homeostatic mechanism that integrates a complex network of signaling pathways to maintain cellular survival and function. Our study demonstrates that modulation of either *SLC29A1* or *SLC29A3* alone is sufficient to significantly alter autophagy flux, thereby shifting the autophagic balance. Interestingly, we observed a reciprocal regulation of *SLC29A1* and *SLC29A3* expression in both stably overexpressing cell lines, *prkaa1 prkaa2* dKO MEFs and to a certain degree, in *slc29a3*^*-/-*^ mice, suggesting a compensatory mechanism aimed at restoring basal autophagic turnover. Furthermore, our findings reveal a bidirectional relationship: while ENTs modulate autophagy, perturbations in autophagy also influence ENT expression. Specifically, pharmacological activation of autophagy upregulates *SLC29A1* and downregulates *SLC29A3*, whereas autophagy inhibition produces the opposite effect. This suggests a dynamic feedback loop in which ENT expression and autophagic activity are co-regulated as part of an adaptive cellular response which warrants further investigations.

## Materials and methods

### Antibodies

Two goat polyclonal antibodies targeting the carboxyl (C20; sc-48147) and amino (N18; sc-48149) termini of human *SLC28A3*/ENT3 (HsSLC28A3/ENT3) were obtained from Santa Cruz Biotechnology. A rabbit polyclonal antibody against the third intracellular loop of human SLC29A3 (as previously described) [30] and another rabbit polyclonal anti-human *SLC29A3* antibody (PA5-38039) were additionally used (Thermo Fisher Scientific). Rabbit polyclonal anti-human *SLC28A1*/ENT1 antibody (ab135756) was purchased from Abcam. Rabbit monoclonal antibodies against MAP1LC3B/LC3B (3868S), phospho-MTOR (5536S), total MTOR (2983S), phospho-RPS6KB/S6K (9234S), total RPS6KB/S6K (2708S), phospho-EIF4EBP1/4E-BP1 (2855S), total EIF4EBP1/4E-BP1 (9644S), phospho-ULK1 (Ser555, 5869S; Ser757, 14202S), total ULK1 (6439S), phospho-ACACA/ACC (11818S), and rabbit polyclonal antibodies against phospho-PRKAA/AMPK (2531S), total PRKAA/AMPK (2532S), and total ACACA/ACC (3662S) were obtained from Cell Signaling Technology. Mouse monoclonal anti-SQSTM1/p62 antibody (610833) was from BD Biosciences. ACTB/β-actin antibody was from Sigma (A1978), and anti-GFP/YFP antibody (sc-9996) from Santa Cruz Biotechnology. Horseradish peroxidase (HRP)-conjugated secondary antibodies (anti-rabbit IgG, anti-mouse IgG, and anti-goat IgG) were purchased from Bethyl Laboratories (A120-501P, A90-217P, A50-101P). Alexa Fluor 488 and 594-conjugated secondary antibodies (anti-rabbit, anti-mouse, and anti-goat) were obtained from Invitrogen/Thermo Fisher Scientific (A-11008, A-11059, A-11055, A-11012, A-11062, A-11058).

### Plasmids

GIPZ lentiviral shRNA plasmid sets targeting *SLC29A3* (RHS4531-EG55315) and *SLC29A1* (RHS4531-EG2030), along with non-targeting controls and lentiviral packaging kits, were purchased from Dharmacon (S-004000-01, TLP5917). Human *SLC29A1* and *SLC29A3 were* cloned into the peYFP-C1 vector (Clontech, 6006–1) and designated as peYFP-*SLC29A1* and peYFP-*SLC29A3*, respectively.

### Chemicals and reagents

Chemical reagents including chloroquine, adenosine, AICAR, 5′-iodotubercidin, and dorsomorphin were purchased from Sigma (C6628, A4036, 123,040, I100, P5499). Rapamycin was obtained from Enzo Life Sciences (BML-A275), and torin1 from Selleck Chem (S2827). The BCA protein assay kit was from Thermo Fisher Scientific (23225). Fetal bovine serum, dialyzed fetal bovine serum, and horse serum were sourced from HyClone Laboratories (SH30071.03, SH30079.03, SH3007404). Fluorescent antifade mounting reagent and penicillin-streptomycin were obtained from Thermo Fisher Scientific/Molecular Probes (P36965, 15–140-148). High-glucose and glucose-free DMEM media were purchased from Thermo Fisher Scientific (11–965-092, 31–053-028). L-methionine sulfone for cell lysis was procured from Sigma-Aldrich (LM; M0876).

### Cell culture

HEK 293 (CRL-1573), and HeLa (CCL-2) cells were obtained from the American Type Culture Collection (ATCC). Both cell lines were cultured in Dulbecco’s Modified Eagle Medium (DMEM; [Thermo Fisher Scientific, 11–965-092]) supplemented with 5% FBS and 1% penicillin-streptomycin solution (100X; Corning Life Sciences, 30–002-CI). *prkaa1 prkaa2* dKO MEFs were cultured as described previously [37]. Lentiviral packaging cell line HEK 293T/17 was obtained from ATCC (CRL-11268). Mouse lung endothelial cells (MLECs) were isolated using previously described method [90]. Briefly, mice lung tissues were harvested, minced, and digested in 3 mg/ml collagenase I (Stem Cell Technologies, 07415) for 45 min and filtered through a 70 µm cell strainer. Cells were centrifuged and incubated with PECAM1/CD31-conjugated dynabeads (Themo Fisher Scientific, 11155D). When cells become confluent, second sorting was performed using ICAM2-conjugated dynabeads (Themo Fisher Scientific, 14311D). Finally, cells were seeded in 12-well plates for experiments [90]. For isolating mouse bone marrow-derived macrophages (BMDM), a method used by Toda et al. [91] was followed. The mouse femur and tibia were extracted ensuring all connective tissue is removed from the bones. The bones were cut open at the ends and flushed using a 27-gauge needle and a 1-ml syringe filled with 10% FBS containing DMEM medium. The flushed cells were cultured in DMEM supplemented with 40 ng/ml CSF1/M-CSF (Stem Cell Technologies, 78,057.1) for 7 days for differentiation into macrophages. The cells were harvested and used for experiments [91].

### Transfection

Transient transfections were performed using Fugene 6 transfection reagent (Roche, F6-1000) according to the manufacturer’s instructions. Briefly, the reagent is mixed with plasmid expression vectors (for YFP, *SLC29A1*-YFP, *SLC29A3*-YFP) in a serum-free medium, incubated the mixture to form a complex, and then added the complex to cells in culture dropwise. After 24 h, transfection efficiency is measured by quantifying YFP fluorescence.

### Lentiviral transduction

Lentiviral shRNA vectors (Horizon Discovery) targeting *SLC29A1* and *SLC29A3* were used to transfect packaging HEK293T cells to generate lentiviral particles using the Dharmacon Trans-Lentiviral shRNA Packaging Kit (TLP5912). The particles were harvested, titered and used to transduce HEK293 cells. The puromycin (2 µg/ml)-resistant cells were assessed for *SLC29A1* and *SLC29A3* protein expression by western blotting analysis in reference to cells transduced with GIPZ control shRNA vector; ACTB was used as the internal loading control [37].

### Real-time PCR

Total RNA was extracted using the Omega E.Z.N.A. Total RNA Kit (Omega Bio-Tek, R6834-00S). cDNA was synthesized from 1 μg of RNA using the RevertAid RT Reverse Transcription Kit (Thermo Fisher Scientific, K1691). TaqMan probes for *SLC29A1* (Hs00191940_m1), *SLC29A3* (Hs00983219_m1), and *GUSB* (Hs00939627_m1) were obtained from Applied Biosystems. Real-time PCR was performed as previously described [37].

### Immunoblotting

Cells or tissues were lysed in TNE buffer prepared in-house (10 mM Tris-HCl, pH 8.0, 0.5% Nonidet *p*-40 [Thermo Fisher Scientific, J19628.K2], 1 mM EDTA, 2 mM PMSF [MilliporeSigma, 52,332], protease and phosphatase inhibitors [Thermo Fisher Scientific, A32955, A32957] on ice for 15 min. Protein concentrations were determined using the BCA assay (Thermo Fisher Scientific, PI23227). Lysates (20–50 μg) were resolved by 10–12% SDS-PAGE and transferred to PVDF membranes (Bio-Rad, 1,620,177). Membranes were blocked in 5% BSA in TBST (constituted in house), incubated with primary antibodies overnight at 4°C, followed by secondary antibody incubation for 2 h. Detection was performed using ECL substrate (Thermo Fisher Scientific, 32,106) and imaged with a Bio-Rad ChemiDoc Touch system. Band intensities were quantified using ImageJ 1.53t and normalized to ACTB.

### Co-immunoprecipitation

HEK293 cells were lysed in CHAPS buffer (20 mM Tris, pH 7.4, 137 mM NaCl, 2 mM EDTA, 10% glycerol, 2% CHAPS [Thermo Fisher Scientific, 28,299]) for 3 h at 4°C. Lysates were incubated overnight with rabbit polyclonal antibodies (1:80; Cell Signaling Technology, 3495, 2827). Protein A Sepharose beads (Amersham Biosciences, 28,944,006) were added for 2 h. Beads were washed twice with 137 mM NaCl CHAPS buffer and twice with 274 mM NaCl CHAPS buffer. Immunoprecipitates were analyzed by SDS-PAGE and immunoblotting.

### Confocal microscopy

Cells were seeded on glass coverslips, fixed with 4% paraformaldehyde for 10 min, permeabilized, and blocked. Primary antibodies were applied, followed by Alexa Fluor 488 or 594-conjugated secondary antibodies. Coverslips were mounted using antifade reagent (Molecular Probes) and imaged using an Olympus Fluoview FV1000 confocal microscope. LC3B puncta were counted using ImageJ.

### Transmission electron microscopy

Liver tissues or HEK293 cells were fixed in 2% glutaraldehyde for 3 h, post-fixed in 1% osmium tetroxide, dehydrated in ethanol, and embedded in Eponate 12 resin (Thermo Fisher Scientific, NC1261266). Ultrathin sections were cut using a Reichert Ultracut E ultramicrotome, stained with 2% uranyl acetate and Reynolds lead citrate, and imaged using a FEI Technai Spirit electron microscope at 80 kV.

### Ado transport assay

Cells were washed with ice-cold phosphate-buffered saline (PBS; Corning 21–040-CV) and incubated in 1× sodium buffer containing 30 µM adenosine (MilliporeSigma, A9251, with or without 10 µM NBMPR (MilliporeSigma, N2255), for 30 min. Cells were then trypsinized, washed, and incubated again in 1× sodium buffer for 30 min. Cell pellets were collected for intracellular nucleoside quantification, and supernatants were retained for adenosine efflux analysis. Intracellular nucleoside extraction was performed as previously described [92]. Briefly, cells were lysed in 500 µl of pre-chilled Extraction Buffer 1 (1 mM LMS, 99.7% methanol) and incubated on ice for 10 min with gentle vortexing. Subsequently, 400 µl of pre-chilled chloroform was added, followed by 200 µl of pre-chilled ultrapure water. The mixture was rotated at 4°C for 15 min and centrifuged at 10,000 × g for 5 min at 4°C. The upper aqueous phase (350 µl) was transferred to a preconditioned ultrafiltration device (Amicon® Ultra, 3 kDa MWCO; MilliporeSigma, UFC200324) and centrifuged at 16,000 × g for 1 h at 4°C. The filtrate was collected, dried using a SpeedVac concentrator, and reconstituted in 25 µl of ultrapure water. After centrifugation at 16,000 × g for 10 min at 10°C, 20 µl of the supernatant was transferred to a fresh tube for LC-MS/MS analysis. Samples were analyzed immediately or stored at −20°C with sealed caps to prevent contamination. For extracellular Ado extraction, the supernatant (1× sodium buffer containing effluxed Ado) was dried and reconstituted in ultrapure water using the same procedure as for intracellular samples. [^3^H]Ado transport assay was performed as described previously [30–32].

### Subcellular fractionation for lysosome purification

Enriched lysosomes from the mouse tissues and HEK293 cells stably transfected with *GIPZ* shRNA and *SLC29A3* shRNA (B05 clone) were obtained as by differential centrifugation, followed by density gradient centrifugation and calcium precipitation (Sigma-Aldrich, LYSISO1) as described before [37]. To determine the enrichment and recovery of lysosomes, the total homogenate and different lysosomal fractions were compared for the protein concentration (using BCA protein assay) and acid phosphatase activity (Sigma-Aldrich, CS0740), while the intactness of the lysosomes was assessed using the neutral red dye following manufacturer’s protocol (Sigma-Aldrich, CS0740).

### LC-MS/MS analysis of Ado

Quantification of nucleosides was performed using a Thermo TSQ Quantiva triple quadrupole mass spectrometer coupled with a Vanquish UHPLC system. Chromatographic separation was achieved using a Waters XBridge BEH C18 column (2.1 × 100 mm, 2.5 µm) maintained at 45°C. The mobile phases consisted of (A) water with 0.2% formic acid and (B) methanol with 0.1% formic acid. The gradient elution profile was as follows: 0–1 min, 0.1% B; 1–2 min, 10–45% B; 2–4 min, 45–95% B; 4–6 min, re-equilibration at 0.1% B. The flow rate was 0.450 ml/min, and the injection volume was 2 µl. The mass spectrometer was operated in positive ion mode using a heated electrospray ionization (HESI) source. Instrument parameters used were: spray voltage, 3.5 kV; capillary temperature, 350°C; sheath gas, 40 arbitrary units; auxiliary gas, 10 arbitrary units; scan range, m/z 100–500; resolution, 70,000. Raw data were processed using Thermo Xcalibur or Skyline software. Extracted ion chromatograms (EICs) were integrated, and peak areas were normalized to an internal standard (10 µM 2′-chlorodeoxyadenosine, 2-CdA) or total ion count, as appropriate. Quantification was performed using a standard curve generated from HPLC-grade adenosine (10 nM-100 µM; Millipore Sigma, K10025A).

### Mouse husbandry

B6.129X1-*Slc29a1*^*tm1Msg*^/J (017739) mice deficient for *slc29a1* were procured from The Jackson Laboratory (The Jackson Laboratory, Bar Harbor, ME, USA). B6;129S5-*Slc29a3*^*tm1Lex*^/Mmucd mice mutant for *Slc29a3* were obtained from the MMRRC repository (UC Davis, CA). All the mice were housed in the institutional facility and maintained at an ambient temperature of 20–22°C with a 12-h light/dark cycle and free access to standard rodent chow and water. For genotyping, tail clips were obtained at 3 weeks of age, and DNA was isolated by proteinase K digestion followed by the isopropanol/ethanol precipitation method. Genotyping of *slc29a1*^*-/-*^ mice was performed using PCR (JAX Protocol 24,539, Version 1.2) with the following primers: Common Forward (14056) − 5’GTT GGG ACC TCT GTC CCT CT 3’; WT Reverse (14055) − 5’ GGT TCT GTC TCC CGT GTC AT 3’; Mut Reverse (14057) − 5’ AAT GCT GGG GAG TAG CAA AG 3.’ Genotyping of *slc29a3*^*-/-*^ mice was performed using PCR with the following primers: WT Forward (DNA426-5) − 5’ GCCAGAGGATCGCTTCA 3’; WT Reverse (DNA426-6) 5’ GACAACAGCAGGCAATCTAAA 3’; Mut Forward (DNA426-22) − 5’ ATGCAAATGGCCTCCCTCAAGTACC 3’; Mut Reverse (PuroJA) − 5’ GAGGAAATTGCATCGCATTGTCT 3.’ PCR cycle used included initial denaturation (94 °C/2 min) followed by ten cycles of denaturation (94°C, 15 s), annealing (65°C, 15 s -touchdown −0.5°C/cycle decrease), elongation (72°C, 45 s) and 30 standard cycles at 60°C annealing temperature and final extension (72°C/2 min). These conditions generate product sizes of 393 and 330 bp for WT and mut *Slc29a1* and 415 and 284 bp for WT and mut *Slc29a3*, respectively. Mutant lines were maintained using heterozygous ×heterozygous breeding strategy, and the mutant lines were backcrossed with WT mice after 10 generations. For each experiment, *n* = 3 mice per group were euthanized using CO_2_ asphyxiation and organs were snap frozen in liquid nitrogen for western blotting and LC-MS/MS experiments. To accomplish uniformity in animal use, we employed female mice for all the animal experiments. All procedures were approved by the Institutional Animal Care and Use Committee (IACUC) at The Ohio State University.

### MEF isolation

Mouse embryonic fibroblasts (MEFs) were isolated as previously described [93]. Briefly, embryos were harvested from pregnant dams (15–20 days gestation) of wild type, *slc29a1*^−/−^ , and *slc29a3*^−/−^ genotypes. Embryos were washed in PBS, and placental tissues were removed. The head and limbs were excised, followed by evisceration. The remaining embryo trunks were finely minced in 1 ml of trypsin and transferred to a sterile dish. Tissues were incubated at 37°C for 30 min with vigorous pipetting every 10 min to facilitate dissociation. Following incubation, the cell suspension was centrifuged at 300 rpm for 5 min. The resulting cell pellet was resuspended in MEF culture medium (DMEM supplemented with 10% fetal bovine serum and penicillin-streptomycin) and plated in T75 flasks. Cells were cultured until confluency, typically within 3 - 4 days, before being used for immunoblotting and immunostaining.

### Microarray analysis

Mouse liver tissue samples were collected from WT and *slc29a3*^−/−^ mice and immediately snap-frozen in liquid nitrogen. Frozen tissues were stored at −80°C until RNA extraction. Total RNA was isolated from 30 mg of liver tissue using the Qiagen RNeasy Mini Kit (Qiagen, 74,106), following the manufacturer’s protocol for tissue samples. The RNA samples were submitted to Genomics Core of Nationwide Children’s Hospital (Columbus, OH) for microarray analysis. Briefly, the RNA integrity was assessed using an Agilent 2100 Bioanalyzer, with samples showing RNA integrity number (RIN) >7.0 included for analysis. 100–500 ng of total RNA for each sample was used to synthesize biotin-labeled complementary RNA (cRNA) using the Illumina TotalPrep RNA Amplification Kit (Thermo Fisher Scientific AMIL1791). The labeled cRNA (750 ng) was then hybridized to Illumina MouseRef-8 v2.0 Expression BeadChips for 16 h at 58°C with constant agitation. Following hybridization, chips were washed and stained with streptavidin-Cy3 using standard Illumina protocols. Arrays were scanned using the Illumina iScan^TM^ System, and raw intensity data were extracted using GenomeStudio software 2.0.5. Quality control checks were performed to assess overall signal intensity, background levels, and internal hybridization controls. Subsequent data processing and normalization were performed in Lumi, an open-source R package, applying variance-stabilizing transformation and robust spline normalization. Differential gene expression (DEG) analysis was conducted using the Limma-Voom R package, with significance thresholds set at an adjusted *p* < 0.05 and a fold change ≥ 2. Further analysis was performed using ShinyGO 0.77 to generate dot plots and networking plots of top pathways enriched in the obtained dataset. The genes were sorted by cross-referencing with the Autophagy Database gene lists [63] sorted based on FDR cutoff and edge cutoff.

### Statistical analysis

Data are presented as mean ± SEM. Statistical significance was determined using Student’s t-test when comparing 2 groups and ANOVA when comparing more than 2 groups in GraphPad Prism 10.4.1 (GraphPad Software Inc., CA). Unpaired parametric t test was used with Welch’s correction, no equal SD assumption and 95% confidence interval (CI), where *p* < 0.05 was considered statistically significant. An ordinary ANOVA test with Dunnett’s test as a post hoc analysis was used for multiple comparisons at 90% CI and *p* < 0.05 was considered statistically significant.

## Supplementary Material

SupplementaryData R4.docx

## Data Availability

The authors confirm that the data supporting the findings of this study are available within the article and its supplementary materials. Microarray data from the SLC29A3/ENT3 Knockout Mouse Liver Whole Transcriptome are available in the ArrayExpress database at the European Bioinformatics Institute (EMBL-EBI) under accession number E-MTAB-15905.

## Abbreviations

ADK: adenosine kinase,
Ado: adenosine,
AMPK: AMP-activated protein kinase,
CNT: concentrative nucleoside transporter,
CQ: chloroquine,
EC: extracellular,
ENT: equilibrative nucleoside transporter,
IC: intracellular,
MTOR: mechanistic target of rapamycin kinase,
RAPA: rapamycin
