# Supplementary material for: SLC29A1/ENT1 and SLC29A3/ENT3 differentially regulate autophagy
Source: Autophagy. 2026 Mar 5;22(6):1401–21. doi: 10.1080/15548627.2026.2639407 (PMC13185436; doi:10.1080/15548627.2026.2639407)
Supplement: SupplementaryData R4.docx [file KAUP_A_2639407_SM3193.docx]

SLC29A1/ENT1 and SLC29A3/ENT3 Differentially Regulate Autophagy

Bhawana Bissa^1#^, Tejinder Kaur^1^, Arnav Joshi^1^, and *Rajgopal Govindarajan^1,2^

Supplementary Data

**Table S1.** Top up- and downregulated autophagy genes in *slc29a3*^-/-^ mice (based on a threshold of fold-change ≥2 and p<0.05).

| **S. no.** | **Gene Symbol^#^** | **Fold-change wrt WT** | **p value** |
| --- | --- | --- | --- |
|  | *Ugt2b1* | -39.7973 | 0.002484 |
|  | *Ugt2a3* | -15.8213 | 0.005199 |
|  | *Ugt2b5* | -15.4368 | 0.001437 |
|  | *Ugt2b35* | -15.3983 | 0.011085 |
|  | *Gabarap* | -12.9592 | 0.000148 |
|  | *Ugt2b38* | -9.9818 | 0.000785 |
|  | *Ugt2b36* | -6.48632 | 0.010158 |
|  | *Rps6kb2* | -6.38506 | 0.000311 |
|  | *Sar1b* | -6.38506 | 0.001362 |
|  | *Ugt2b37* | -6.19971 | 0.00087 |
|  | *Cdk18* | -6.00099 | 0.000691 |
|  | *Deptor* | -5.71518 | 0.001798 |
|  | *Deptor* | -5.71518 | 0.001798 |
|  | *Trappc1* | -5.62323 | 0.000186 |
|  | *Ube2n* | -5.5056 | 0.000105 |
|  | *Ppp2r5e* | -4.78759 | 0.002609 |
|  | *Krt8* | -4.77798 | 0.001691 |
|  | *Acbd5* | -4.46481 | 0.00036 |
|  | *Prkaa2* | -4.38021 | 0.001516 |
|  | *Sec24c* | -3.768 | 0.000202 |
|  | *Ppp1ca* | -3.6394 | 0.001853 |
|  | *Vamp7* | -3.4011 | 0.018796 |
|  | *Csnk1g3* | -3.33549 | 0.003913 |
|  | *Mtmr9* | -3.31935 | 0.000118 |
|  | *Mfn2* | -3.24249 | 0.004926 |
|  | *Ppp2r5a* | -3.16345 | 0.007169 |
|  | ***Atg4a*** | **-3.09663** | **0.000107** |
|  | *Ern1* | -3.04892 | 0.005372 |
|  | *Nfe2l1* | -2.9365 | 0.001599 |
|  | *Ulk2* | -2.89086 | 0.001138 |
|  | *Ppp6c* | -2.86831 | 0.00154 |
|  | *Map2k3* | -2.85403 | 0.000555 |
|  | *Mapk3* | -2.6898 | 0.000247 |
|  | *Rab8a* | -2.66919 | 0.002631 |
|  | *Prkaa1* | -2.64433 | 0.00382 |
|  | *Ulk1* | -2.50012 | 0.012457 |
|  | *Camk2b* | -2.45922 | 0.000462 |
|  | *Opa1* | -2.44715 | 0.001318 |
|  | *Pa2g4* | -2.41078 | 0.000847 |
|  | *Tsc2* | -2.40161 | 0.000367 |
|  | *Pink1* | -2.38254 | 0.002976 |
|  | *Map2k4* | -2.35267 | 0.001027 |
|  | *Nbr1* | -2.33044 | 0.004229 |
|  | *Tns2* | -2.31001 | 0.006861 |
|  | *Fkbp8* | -2.30042 | 0.000914 |
|  | *Camk1d* | -2.29915 | 0.030857 |
|  | *Araf* | -2.28802 | 0.001379 |
|  | *Yes1* | -2.28438 | 0.000156 |
|  | *Rab5b* | -2.27048 | 0.000182 |
|  | *Ap2a2* | -2.26263 | 0.002174 |
|  | *Ppp2r5d* | -2.23985 | 0.000179 |
|  | *Ap1m1* | -2.2176 | 0.001896 |
|  | *Mtmr4* | -2.21345 | 0.002325 |
|  | *Pex1* | -2.19208 | 0.003124 |
|  | *Sec23a* | -2.14459 | 0.012073 |
|  | *Sec23a* | -2.14459 | 0.012073 |
|  | *Stx17* | -2.12771 | 0.001516 |
|  | *Vdac3* | -2.0942 | 0.007225 |
|  | *Capn2* | -2.07311 | 0.00066 |
|  | *Afg3l1* | -2.01726 | 0.000663 |
|  | *Sec23b* | -2.00959 | 0.019282 |
|  | *Shc1* | -2.00264 | 0.000473 |
|  | *Grb2* | 2.050534 | 0.004158 |
|  | *Rab10* | 2.085076 | 0.017197 |
|  | ***Stk10*** | **2.091155** | **0.000739** |
|  | *Man2a1* | 2.117556 | 0.036658 |
|  | *Kras* | 2.261531 | 0.001841 |
|  | *Cltc* | 2.510196 | 0.003102 |
|  | *Actr2* | 2.591523 | 0.004115 |
|  | *Tpr* | 2.642494 | 0.000404 |
|  | *Sgk1* | 2.726216 | 0.00077 |
|  | *Smg1* | 2.781186 | 0.000895 |
|  | *Mertk* | 2.784079 | 0.005727 |
|  | ***Prkcd*** | **2.848693** | **0.00259** |
|  | *Rgl1* | 2.890261 | 0.000711 |
|  | *Myd88* | 2.943229 | 0.005827 |
|  | *Stx7* | 3.038165 | 0.000481 |
|  | *Wdfy3* | 3.047656 | 0.000109 |
|  | *Ppp2cb* | 3.057177 | 0.005707 |
|  | *Stk24* | 3.197402 | 0.000996 |
|  | *Axl* | 3.22612 | 0.01035 |
|  | *Smpdl3a* | 3.491628 | 0.011884 |
|  | ***Rel*** | **3.579604** | **0.010555** |
|  | *Wipi1* | 3.717158 | 0.034662 |
|  | *Fyn* | 4.029498 | 0.000441 |
|  | *Sik1* | 4.839305 | 0.018415 |
|  | *Sesn1* | 5.286453 | 0.025377 |
|  | *Ctsl* | 5.606498 | 0.00683 |
|  | *Actr3* | 6.362087 | 0.000486 |
|  | *Nfkbia* | 7.368648 | 0.008136 |
|  | *Arntl* | 9.056163 | 0.009813 |
|  | ***Lyn*** | **12.13078** | **0.000428** |
|  | *Vim* | 16.67367 | 0.00086 |
|  | *Slc3a1* | 35.9869 | 0.025789 |
|  | *Ddit4* | 81.88345 | 0.001174 |

^#^Genes in **bold** denote genes commonly enriched in both *slc29a1*^-/-^ and *slc29a3*^-/-^

**Table S2.** Top up- and downregulated autophagy genes in *slc29a1*^-/-^ mice (based on a threshold of fold-change ≥2 and p<0.05).

| **S. no.** | **Gene Symbol^#^** | **Fold-change wrt WT** | **p value** |
| --- | --- | --- | --- |
|  | *Ank1* | -4.4478 | 0.0367 |
|  | *Ankrd44* | -4.0221 | 0.035 |
|  | *Arhgap42* | -3.6426 | 0.0037 |
|  | ***Atg4a*** | **-2.7936** | **0** |
|  | *Aurka* | -2.7054 | 0.0079 |
|  | *Bnip3* | -2.5475 | 0.0098 |
|  | *Camk2a* | -2.5459 | 0.0006 |
|  | *Capn1* | -2.392 | 0.0003 |
|  | *Capn6* | -2.2934 | 0.0003 |
|  | *Casp3* | -2.23 | 0.0475 |
|  | *Cdk1* | -2.1302 | 0.0009 |
|  | *Cdk2* | -2.0967 | 0.002 |
|  | *Celf2* | -2.0798 | 0 |
|  | *Ctse* | -2.012 | 0.0225 |
|  | *Des* | 2.0081 | 0.0029 |
|  | *E2f1* | 2.0176 | 0.0068 |
|  | *E2f2* | 2.0281 | 0.0006 |
|  | *E2f3* | 2.0318 | 0.009 |
|  | *Eef1a2* | 2.0339 | 0.0015 |
|  | *Eif2ak1* | 2.0463 | 0.0044 |
|  | *Exoc6* | 2.0728 | 0.0006 |
|  | *Fgr* | 2.0778 | 0.0009 |
|  | *Glipr2* | 2.088 | 0.0004 |
|  | *Hck* | 2.2087 | 0.0057 |
|  | *Hspa1a* | 2.2808 | 0.0067 |
|  | *Ikbke* | 2.2843 | 0.0022 |
|  | ***Lyn*** | **2.3185** | **0.0005** |
|  | *Man2b1* | 2.3501 | 0.0017 |
|  | *Map4k1* | 2.3655 | 0.0044 |
|  | *Map4k2* | 2.3724 | 0.0008 |
|  | *Mapk13* | 2.4063 | 0.0019 |
|  | *Mapk14* | 2.4929 | 0.0004 |
|  | *Napsa* | 2.531 | 0.0001 |
|  | *Ncoa4* | 2.5735 | 0.0001 |
|  | *Nfkbid* | 2.6112 | 0.0033 |
|  | *Nuak2* | 2.6243 | 0.0025 |
|  | *Pik3cd* | 2.6243 | 0.0037 |
|  | *Pik3cg* | 2.6392 | 0.0036 |
|  | *Pip4k2a* | 2.6727 | 0 |
|  | *Pip5k1b* | 2.6766 | 0.0018 |
|  | *Plcl2* | 2.686 | 0.0077 |
|  | *Ppp2r3a* | 2.7666 | 0.0002 |
|  | *Prkab1* | 2.7732 | 0.0023 |
|  | *Prkcb* | 2.8032 | 0.0081 |
|  | ***Prkcd*** | **2.8546** | **0.0015** |
|  | *Rcan1* | 2.8573 | 0.0014 |
|  | ***Rel*** | **2.8806** | **0.0061** |
|  | *Rgs19* | 2.9004 | 0.0063 |
|  | *Rps6ka1* | 2.9439 | 0.0082 |
|  | *Ryr1* | 3.0682 | 0.0036 |
|  | *Slc1a1* | 3.3509 | 0.0002 |
|  | *Slc1a4* | 3.6815 | 0.0145 |
|  | *Slc7a11* | 3.724 | 0.0024 |
|  | *Slc7a5* | 3.8681 | 0.0027 |
|  | *Slc7a8* | 3.901 | 0.0022 |
|  | ***Stk10*** | **3.9503** | **0.0008** |
|  | *Stk38l* | 3.9995 | 0.0028 |
|  | *Stxbp1* | 4.1004 | 0.0044 |
|  | *Stxbp2* | 4.1263 | 0.0023 |
|  | *Tmem173* | 4.9977 | 0.0042 |
|  | *Tnfaip2* | 5.5283 | 0.0006 |
|  | *Wdfy4* | 6.3477 | 0.0047 |

^#^Genes in **bold** denote genes commonly enriched in both *slc29a1*^-/-^ and *slc29a3*^-/-^

**B**


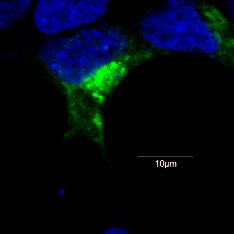


**10 μm**


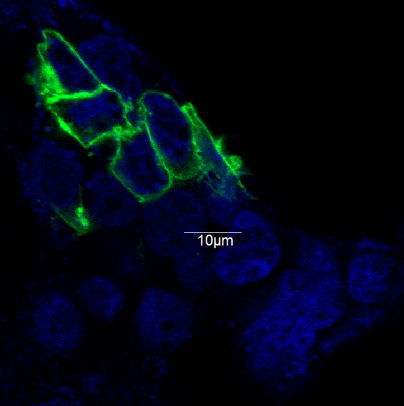


**10 μm**

**SLC29A1**

**SLC29A3**

**A**

**Figure S1.** *SLC29A1* and *SLC29A3* expression analysis in HEK293 cells. (**A**) Endogenous mRNA expression of *SLC29A1* and *SLC29A3* in HEK293 cells analyzed by real-time qPCR. Data are presented as mean ΔCt ± SEM. Ct values for target and reference genes are indicated in italics. A low Ct value denotes high target gene expression and *vice versa*. (**B**) Representative immunofluorescence images of HEK293 cells expressing endogenous SLC29A1 and SLC29A3. Scale bar: 10 μm.


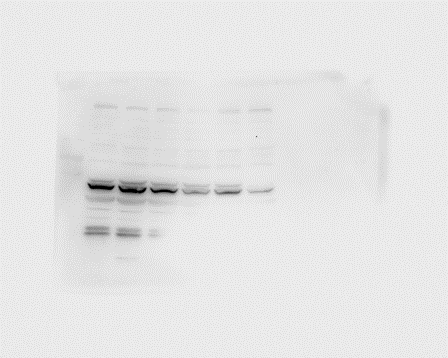

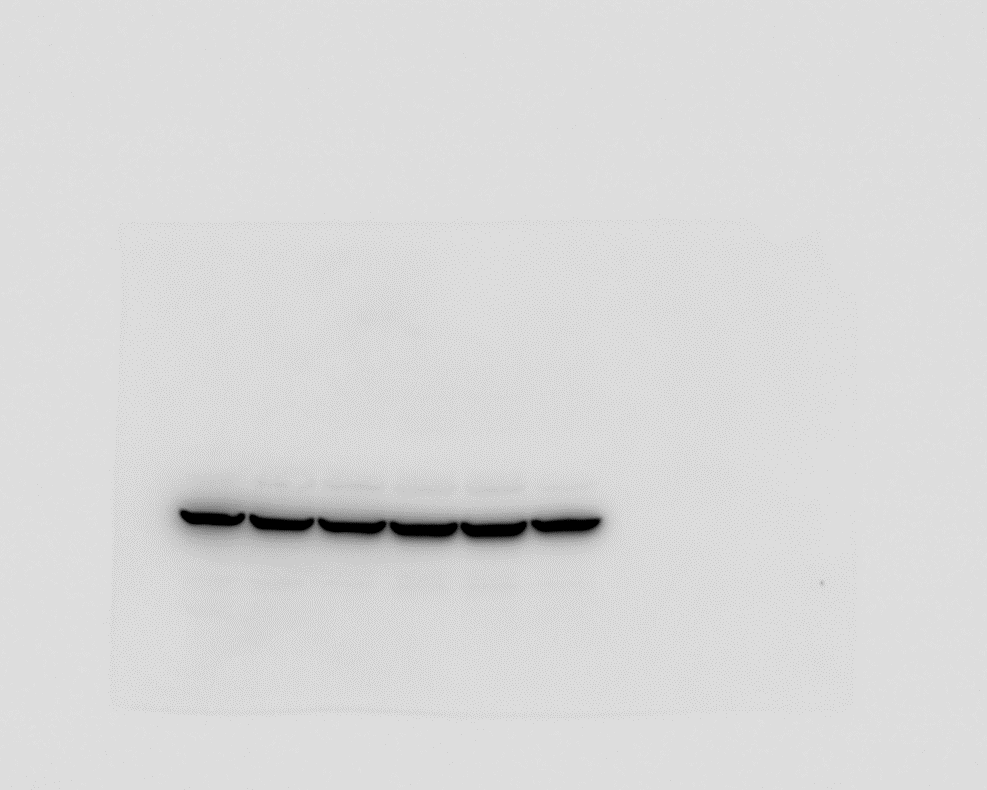


**Gipz D01 D02 D03 D04 D05**

SLC29A1 55 kDa

ACTB

-40

-55

-40

SLC29A1:ACTB 0.9 0.9 0.8 0.4 0.5 0.2

kDa

**Figure S2.** Western blotting analysis of SLC29A1 expression in HEK293 cells transfected with control *GIPZ* shRNA or *SLC29A1*-targeting shRNAs. ACTB was used as a loading control.

**Figure S3.** Quantification of LC3B puncta was performed in 50 cells per condition using ImageJ (n = 3). Data are presented as mean ± SEM. *p < 0.05.

**Figure S4.** HEK293 cells transfected with peYFP, SLC29A1-YFP, or SLC29A3-YFP were analyzed for expression of *ATG5*, *ATG7*, *ATG12*, *ATG16l1*, and *BECN1* by qPCR. Data are presented as 2^–ΔΔCt^ relative to control. Individual bars are compared to respective controls (peYFP or GIPZ), and lines indicate comparisons between SLC29A1 and SLC29A3. *p < 0.05.


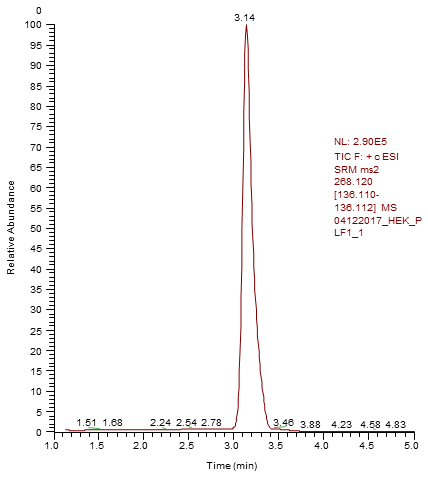


**B**

**A**

**Figure S5.** Ado quantification using LC-MS/MS. (**A**) LC-MS/MS chromatogram for Ado standard (1000 ng/ml). (**B**) Linear calibration curve was plotted for Ado over the range 7.81-1000 ng/ml.

**Figure S6.** Densitometric quantification of SQSTM1 levels in tissues of WT, *slc29a1*^-/-^ and *slc29a3*^-/-^ mice, normalized to ACTB (n = 3). Data are presented as mean ± SEM. *p < 0.05, **p ≤ 0.01, ***p ≤ 0.001.
